# The improved thermal efficiency of Prandtl–Eyring hybrid nanofluid via classical Keller box technique

**DOI:** 10.1038/s41598-021-02756-4

**Published:** 2021-12-07

**Authors:** Wasim Jamshed, Dumitru Baleanu, Nor Ain Azeany Moh Nasir, Faisal Shahzad, Kottakkaran Sooppy Nisar, Muhammad Shoaib, Sohail Ahmad, Khadiga Ahmed Ismail

**Affiliations:** 1 Department of Mathematics, Capital University of Science and Technology (CUST), Islamabad, 44000 Pakistan; 2 Institute of Space Sciences, 077125 Magurele-Bucharest, Romania; 3 Department of Medical Research, China Medical University Hospital, China Medical University, Taichung, 40447 Taiwan; 4 Department of Mathematics, Centre for Defence Foundation Studies, Universiti Pertahanan Nasional Malaysia, Kem Sungai Besi, 57000 Kuala Lumpur, Malaysia; 5 Department of Mathematics, College of Arts and Sciences, Prince Sattam Bin Abdulaziz University, Wadi Aldawaser, 11991 Saudi Arabia; 6 Department of Mathematics, COMSATS University Islamabad, Attock Campus, Attock, Pakistan; 7 Centre for Advanced Studies in Pure and Applied Mathematics (CASPAM), Bahauddin Zakariya University, Multan, 60800 Pakistan; 8 Department of Clinical Laboratory Sciences, College of Applied Medical Sciences, Taif University, P.O. Box 11099, Taif, 21944 Saudi Arabia; 9 Department of Mathematics, Cankaya University, Balgat, 06530 Turkey

**Keywords:** Mathematics and computing, Physics

## Abstract

Prandtl–Eyring hybrid nanofluid (P-EHNF) heat transfer and entropy generation were studied in this article. A slippery heated surface is used to test the flow and thermal transport properties of P-EHNF nanofluid. This investigation will also examine the effects of nano solid tubes morphologies, porosity materials, Cattaneo–Christov heat flow, and radiative flux. Predominant flow equations are written as partial differential equations (PDE). To find the solution, the PDEs were transformed into ordinary differential equations (ODEs), then the Keller box numerical approach was used to solve the ODEs. Single-walled carbon nanotubes (SWCNT) and multi-walled carbon nanotubes (MWCNT) using Engine Oil (EO) as a base fluid are studied in this work. The flow, temperature, drag force, Nusselt amount, and entropy measurement visually show significant findings for various variables. Notably, the comparison of P-EHNF's (MWCNT-SWCNT/EO) heat transfer rate with conventional nanofluid (SWCNT-EO) results in ever more significant upsurges. Spherical-shaped nano solid particles have the highest heat transport, whereas lamina-shaped nano solid particles exhibit the lowest heat transport. The model's entropy increases as the size of the nanoparticles get larger. A similar effect is seen when the radiative flow and the Prandtl–Eyring variable-II are improved.

## Introduction

Liquid mechanics' limits are defined by the thin fluid or liquid layer in contact with the pipe's or an aircraft wing's surface. In the boundary layer, shear forces can damage the liquid. Given that the fluid is in touch with the surface, a range of speeds exists between the maximum and zero boundary layer speeds. Limits on the trailing edge of an aeroplane wing, for example, are smaller and thicker. A thickening of the flow occurs at the front or upstream end of these boundaries. In 1904, Prandtl proposed the concept of boundary layers to describe the flow behaviour of viscous fluid near a solid barrier (see Aziz et al.^1^). Using the Navier Stoke equations, Prandtl constructed and inferred boundary layer equations for large Reynolds number flows. As a necessary simplification of the original Navier–Stokes equation, the boundary layer theory equations were critical. Studying wall jets, free jets, fluid jets, flow over a stretched platform/surface, and inductive flow from a shrinking plate helps develop the equations for these phenomena. Boundary layer equations are often solved using a variety of boundary conditions that are specific to a given physical model. For a magnetohydrodynamics (MHD) fluid flow with gyrotactic microorganisms, Sankad et al.^2^ found that the magnetic and Peclet numbers may be utilised to reduce the thermal boundary layer thickness. After that, Hussain et al.^3^ discovered that the thickness of the thermal boundary layer increases as a Casson liquid flows towards the growing porous wedge due to convective heat transfer. The literature has several experiments with various physical parameter impacts on boundary layer flow^4–6^ and multiple liquids^7,8^.

A hybrid nanofluid is now attracting the attention of many researchers. Hybrid nanofluids are cutting-edge nanofluids that combine two different types of nanoparticles in a single fluid. The thermal properties of the hybrid nanofluid are better than those of the primary liquid and nanofluids. In machining and manufacturing, hybrid nanofluids are commonly utilised in solar collectors, refrigeration, and coolants. According to Suresh et al.^9^, copper nanoparticles in the alumina matrix mixed at most modest and sufficient levels may preserve the hybrid nanofluid's strength, first introduced in^9^. Despite having a lower thermal conductivity than copper nanoparticles, alumina nanoparticles have excellent chemical inactivity and stability. Yildiz et al.^10^ developed an equivalence between theoretical and experimental thermal conductivity models for heat transfer performance in hybrid-nanofluid. In comparison to a mono nanofluid, the hybridisation of nanoparticles improved heat transfer at a lower particle percentage (Al_2_O_3_). Waini et al.^11^ investigated a hybrid nanofluid's unsteady flow and heat transfer using a curved surface. As the surface curvature changed, the presence of dual solutions resulted in intensification in the volume percentage of copper nanoparticles. Many years later, Qureshi et al.^12^ investigated the hybrid mixed convection nanofluid's characteristics in a straight obstacle channel. They've discovered that increasing the barrier's radius improves heat transfer by as much as 119%. In addition, the horizontal orientation of the cylinder only supports a heat transfer efficiency of 2.54%. Mabood and Akinshilo^13^ investigated the influence of uniform magnetic and radiation on the heat transfer flow of Cu-Al_2_O_3_/H2O hybrid nanofluid flowing over the stretched surface. Discoveries such as those made at the science fair show how radiation speeds up heat transport while magnetic forces slow it down. Further hybrid nanofluid studies and experiments have been conducted by these researchers^14–24^.

The Cattaneo–Christov heat flux model describes the heat transfer in viscoelastic flows caused by an exponentially expanding sheet. There may be a relationship between thermal relaxation time and the boundaries of this model. Dogonchi and Ganji^25^ researched unstable squeezing MHD nanofluid flow across parallel plates using a Cattaneo–Cristov heat flux model some years ago. The thermal relaxation parameter, they found, slowed heat transfer. Additionally, Muhammad et al.^26^ discovered that when thermal relaxation increased, the fluid temperature decreased. Other researchers have used the Cattaneo–Christov heat flux model to examine fluid flow and determine the physical features that thermal relaxation affects. Scholars like^27–32^ may be found in the literature as examples of this group. Even the temperature of a nanofluid may be reduced by the thermal relaxation parameter, according to Ali et al.^33^. This finding is critical to the contemporary food, medicinal, and aerospace industries. Waqas et al.^34^ introduced mathematical modelling using the Cattaneo–Christov model for hybrid nanofluid flow in a rocket engine. The finding exposed that the temperature is reduced when thermal relaxation and melting parameters vary, but the Biot number increases. Other types of hybrids nanofluid characteristics using the Cattaneo–Christov model have been discussed by Haneef et al.^35^. The vital discovery uncovered an escalation causes shrinkage in wall shear stress in momentum relaxation time. Different encounters were found by Reddy et al.^36^ in the Cattaneo–Christov model problem for hybrid dusty nanofluid flow. It reveals that dusty hybrid nanofluid has a better heat transfer method than hybrid nanofluid.

Nevertheless, a few years back, a new type of fluid was found called hybrid nanofluid, and many researchers have been eager to search for the characteristics of this type of fluid since then. The research for finding the aspect of non-Newtonian hybrid nanofluid also needed to be done. Latterly, Yan et al.^37^ have conducted an investigation towards the rheological behaviour of non-Newtonian hybrid nanofluid for a powered pump. They reported at the highest volume fraction hybrid nanofluid, the viscosity reduced at most 21%. Nabwey and Mahdy^38^ are doing an inclusive exploration of micropolar dusty hybrid nanofluid. The finding indicates that the temperature fluctuation in both the micropolar hybrid nanofluid and dust phases is strengthened by increased thermal relaxation. Several investigations have been carried out for the different types of non-Newtonian hybrid nanofluid, such as aluminium alloy nanoparticles by Madhukesh et al.^39^, MWCNT-Al_2_O_3_/5W50 by Esfe et al.^40^ and ZnO–Ag/H_2_O by He et al.^41^ in the literature. Despite that, only a few research available in the literature investigating the viscoelastic hybrid nanofluid behaviour. Several models can be used to examine the physical properties of the viscoelastic fluid, including the power-law model, the Prandtl fluid model, and the Prandtl–Eyring model. The power-law model predicts the non-linear relationship between deformation rate and shear stress. It has been hypothesised that shear stress is connected to the sine inverse function of deformation rate by the Prandtl model and that it is related to the hyperbolic sine function of deformation rate by the Prandtl–Eyring model. Hussain et al.^42^ have investigated the physical aspect of MHD Prandtl–Eyring fluid flow and reported that at all positions in the flow domain, a substantial rise in momentum transportation had been seen against an increase in the fluid parameter. Rehman et al.^43^ added in the findings that Prandtl–Eyring liquid particles are subjected to drag forces in a flow when their skin friction coefficients are high (or low). A similar discovery has been conveyed by Khan et al.^44^ which the skin friction improves for the Prandtl–Eyring nanofluid. Later, Akram et al.^45^ model a MHD Prandtl–Eyring nanofluid peristaltic pumping in an inclined channel. This study demonstrates that the wall tension and mass parameters have a rising influence on axial velocity, whereas the wall damping parameter has a decreasing impact. Li et al.^46^ have explored the entropy of the Prandtl–Eyring fluid flow model over a rotating cone. The result demonstration the velocity and temperature have been shown to behave differently when the viscosity parameter increases in magnitude. Latest study for the Prandtl–Eyring hybrid nanofluid model being carried out by Jamshed et al.^47^. The outcome was mentioning the entropy upsurged with radiative flux and Prandtl–Eyring parameter.

The famous numerical technique for solving non-linear boundary layer equations in fluid mechanics is derived by Keller and Cebeci^48^ called Keller Box Method (KBM). It is being popularised by Cebeci and Bradshaw^49^. The technique is known for highly accurate and time computation in solving non-linear problems. A lot of investigations of fluid dynamics have been solved using KBM in the literature. Bilal et al.^50^ implemented the KBM for solving Williamson fluid flow towards a cylindrical surface and found the results are comparable with other published results. Similar numerical computation was reported by Swalmeh et al.^51^ in solving the micropolar nanofluid over a solid sphere using KBM. The computed solution being reported as having a good agreement with the solution computed by bvp4c (MATLAB). The KBM is a universal solver since it is proven can solve another type of mathematical modelling, for instance, Carreau fluid model (Salahuddin^52^), micropolar fluid (Singh et al.^53^), viscous fluid model (Bhat and Katagi^54^), Prandtl nanofluid (Habib et al.^55^), MHD nanofluid (Zeeshan et al.^56^) and third-grade nanofluid (Abbasi et al.^57^).

Size and distribution descriptors should be chosen to offer the most significant discrimination for particulate quality concerning specific attributes or characterisation of a manufacturing process, depending on their use. If particle form affects these attributes, the shape and distribution of the particles should be studied in addition to their size. Qualitative terminology like fibres or flakes can be used, or quantitative terms like elongation, roundness, and angularity can also be used. Other quantitative terms include percentages of certain model forms and fractal dimensions. Despite the importance of the particle shape, only a few research can be found in the literature, such as^58–60^. The latest research has been done by Sahoo^61^, which claimed that the particle shapes heavily influence the thermo-hydraulic performance of a ternary hybrid nanofluid. Similar findings have been illustrated by Elnaqeeb et al.^62^ in hybrid nanofluid flow with the impact of suction and stretching surface. Meanwhile, Rashid et al.^63^ suggested that the temperature and Nusselt number profiles demonstrate the sphere shape nanoparticles have superior temperature disturbance and heat transmission on hybrid nanofluid flow with the influence of relevant factors.

A few publications have examined the impact of the porosity material, viscid dissipative flow, Cattaneo–Christov heat flow and thermal radiative flow shape-factor along the elongated surface using nanofluid Tiwari-Das type on P-EHNF entropy generation. However, none of these papers has addressed these issues. In the Tiwari-Das (monotonic model), the fluid, speed, and temperature are all the same. As a result, the model is simpler and easier to solve when using the single-phase technique numerically. However, this technique has the drawback of resulting in numerical effects that differ from experimental results in some cases. Nanoparticle concentrations in this model volume range from 3 to 20%. Numerical results could only mimic the effects of SWCNT-EO, MWCNT-EO hybrids, and conventional nanofluids in this study. Thus, in order to bridge the gap, the current research focuses on the solid–fluid characteristics impacts and the level of chaos in the boundary layer using the Keller-box technique of P-EHNF.

## Flow model formulations

The mathematical flow equations shows the moved horizontal plate with the irregular expanding velocity^64^:

1 $$ U_{w} \left( {x,t} \right) = bx, $$

where $b$ is an original expanding ratio. Sequestered surface heat is ${\yen}_{w} \left( {x,t} \right) = {\yen}_{\infty } + b^{*} x$ and for the suitability, it is presumed to stand at $x = 0$, where $b*,$ ${\yen}_{w}$, where $\yen_{\infty }$ signify the temperature variation amount, heat of surface, and surrounds correspondingly. The plate is supposed to be slippery, and the surface is subjected to a temperature variation.

Primary addition SWCNT nano solid-particles synthesise the hybrid nanofluid in the EO-based liquid at an interaction volume fraction ($\phi_{ST}$) and it is fixed at 0.09 during the examination. MWCNT nano molecules have been extended in combination to obtain a hybrid nanofluid at the concentrated size ($\phi_{MT}$).

### Prandtl–Eyring fluid stress tensor

Prandtl–Eyring fluid stress tensor is given in the following mathematical form (for example, Mekheimer and Ramadan^65^).

$$ \uptau = \frac{{A_{d} {\text{Sin}}^{{ - 1}} \left[ {\frac{1}{{C{\text{ }}}}\left( {\frac{{\partial B_{1} }}{{\partial y}}} \right)^{2}  + \left( {\frac{{\partial B_{1} }}{{\partial y}}} \right)^{{2^{{\frac{1}{2}}} }} } \right]}}{{\left[ {\left( {\frac{{\partial B_{1} }}{{\partial y}}} \right)^{2}  + \left( {\frac{{\partial B_{1} }}{{\partial y}}} \right)^{{2^{{\frac{1}{2}}} }} } \right]}}\left( {\frac{{\partial B_{1} }}{{\partial y}}} \right). $$

Here the curving velocity indicates the mechanisms $\mathop{B}\limits^{\leftarrow}  = \left[ {B_{1} \left( {x,y,0} \right),B_{2} \left( {x,y,0} \right),0} \right]$. $A_{d}$ and ${\text{C }}$ is fluid parameters.

### Suppositions and terms of system

The following principles, as well as the constraints, apply to the flow system:

**Table:** 

2-D laminar time-dependent curving	Domenating-layer approximations
Single phase (Tiwari-Das) scheme	Non-Newtonian P-EHNF
Porous medium	Cattaneo–Christov heat flux
Thermal radiative flow	Viscid dissipative flowing
Nano solid-particles shape-factor	Porousness elongated surface
Slippery boundary constraints	Thermal jump boundary constraints

### Formal model

**Figure 1:**
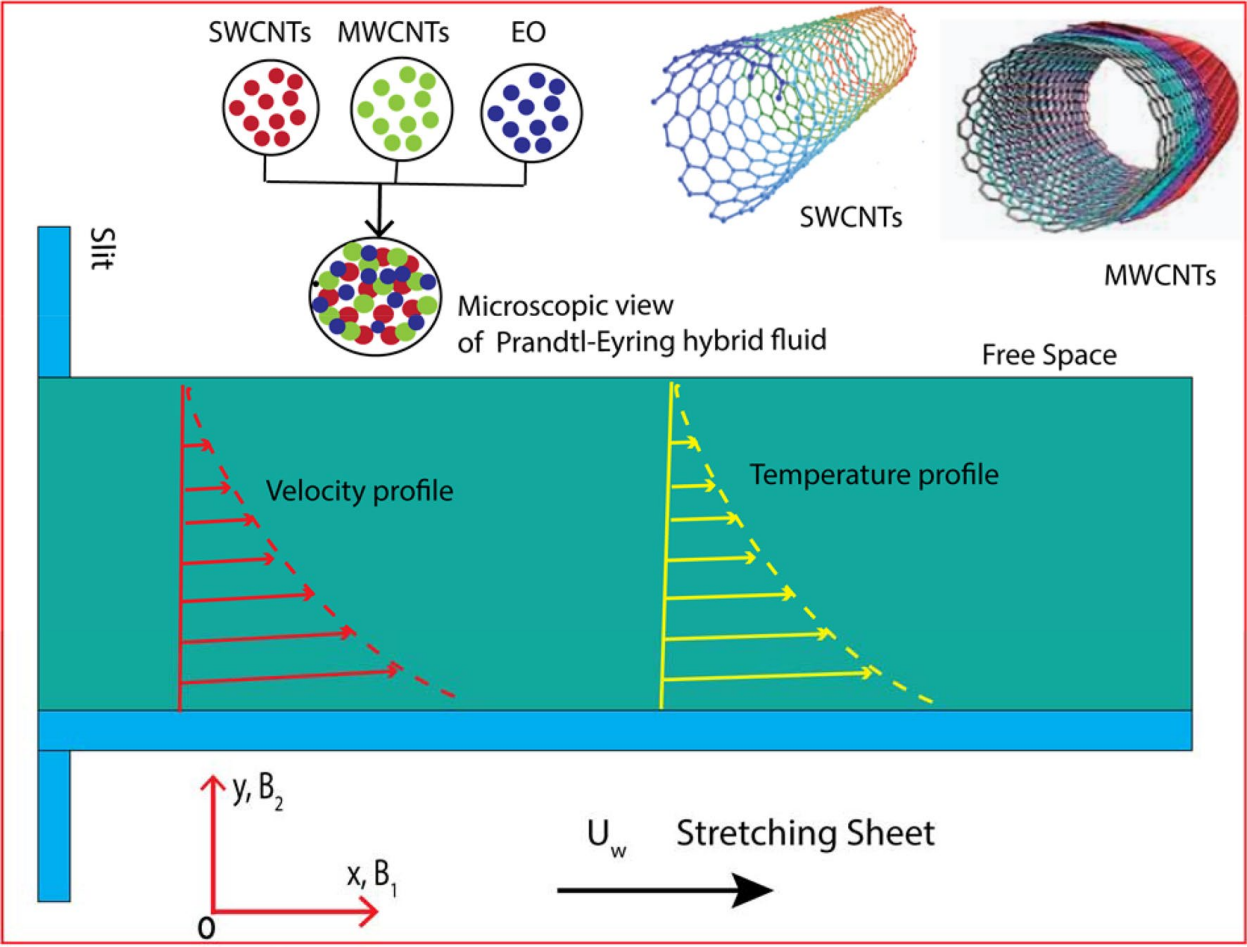
Diagram of the flow model.

The formal (geometric) flowing model is displayed as (Fig. 1):

### Model equations

The constitutive flow formulas^66^ of the viscous Prandtl–Eyring hybrid nanofluid, in combination with a porous material, Cattaneo–Christov heat flux and thermal radiative flow utilising the approximate boundary-layer are

2 $$ \frac{{\partial B_{1} }}{\partial x} + \frac{{\partial B_{2} }}{\partial y} = 0, $$

3 $$ B_{1} \frac{{\partial B_{1} }}{\partial x} + B_{2} \frac{{\partial B_{1} }}{\partial y} = \frac{{A_{{\text{d}}} }}{{C\rho_{hnf} }}\left( {\frac{{\partial^{2} B_{1} }}{{\partial y^{2} }}} \right) - \frac{{A_{{\text{d}}} }}{{2C^{3} \rho_{hnf} }}\frac{{\partial^{2} B_{1} }}{{\partial y^{2} }}\left[ {\left( {\frac{{\partial B_{1} }}{\partial y}} \right)^{2} } \right] - \frac{{\mu_{hnf} }}{{\rho_{hnf} k}}B_{1} , $$

4 $$ \begin{aligned} & B_{1} \frac{\partial \yen}{{\partial x}} + B_{2} \frac{\partial \yen}{{\partial y}} = \frac{1}{{\left( {\rho C_{p} } \right)\kappa_{hnf} }}\left[ {k_{hnf} \left( {\frac{{\partial^{2} \yen}}{{\partial y^{2} }}} \right) + \mu_{hnf} \left( {\frac{{\partial B_{1} }}{\partial y}} \right)^{2} - \frac{{\partial q_{r} }}{\partial y}} \right], \\ & \quad - \delta^{*} \left[ {B_{1} \frac{{\partial B_{1} }}{\partial x}\frac{\partial \yen}{{\partial x}} + B_{2} \frac{{\partial B_{2} }}{\partial y}\frac{\partial \yen}{{\partial y}} + B_{1} \frac{{\partial B_{2} }}{\partial x}\frac{\partial \yen}{{\partial y}} + B_{2} \frac{{\partial B_{1} }}{\partial y}\frac{\partial \yen}{{\partial x}} + B_{1}^{2} \frac{{\partial^{2} \yen}}{{\partial x^{2} }} + B_{2}^{2} \frac{{\partial^{2} \yen}}{{\partial y^{2} }} + 2B_{1} B_{2} \frac{{\partial^{2} \yen}}{\partial x\partial y}} \right]. \\ \end{aligned} $$

the appropriate connection conditions are as follows, which can be located in Aziz et al.^67^:

5 $$ B_{1} \left( {x,0} \right) = U_{w} + N_{\varsigma } \left( {\frac{{\partial B_{1} }}{\partial y}} \right),\quad B_{2} \left( {x,0} \right) = V_{\varsigma } ,\quad - k_{{{\varsigma }}} \left( {\frac{\partial \yen}{{\partial y}}} \right) = h_{\varsigma } \left( {\yen_{w} - \yen} \right), $$

6 $$ B_{1} \to 0,\quad \yen \to \yen_{\infty } \quad as\quad y \to \infty . $$

We formulate the $\yen$ as a fluid heat. Other vital parameters are surface permeability $V_{\varsigma }$, heat transfer coefficient $h_{\varsigma }$, porosity $\left( k \right)$ and heat conductivity of firm $k_{{\varsigma }}$. Physical features identical, Convectional animated surface experienced its heat loss through conductive (Newtonian thermal) and flowing swiftness close to the sheet is comparative to the cut stress exerts in it (slippy form) are deliberate.

### Heat-physical possessions of P-ENF

Nano solid particles dispersed in EO induce improved thermophysical characteristics. The next Table 1 equations summarize P-ENF substance variables^68,69^.

**Table 1:** Thermo-physical features for nano liquids.

Features	Nano liquid
Dynamical viscidness $(\mu )$	$\mu_{nf} = \mu_{f} (1 - \phi )^{ - 2.5}$
Density $(\rho )$	$\rho_{nf} = \left( {1 - \phi } \right)\rho_{f} - \phi \rho_{s}$
Heat capacity $(\rho C_{p} )$	$(\rho C_{p} )_{nf} = \left( {1 - \phi } \right)(\rho C_{p} )_{f} - \phi (\rho C_{p} )_{s}$
Thermal conductivity $(\kappa )$	$\frac{{\kappa_{nf} }}{{\kappa_{f} }} = \left[ {\frac{{\left( {\kappa_{s} + \left( {m - 1} \right)\kappa_{f} } \right) - \left( {m - 1} \right)\phi \left( {\kappa_{f} - \kappa_{s} } \right)}}{{\left( {\kappa_{s} + \left( {m - 1} \right)\kappa_{f} } \right) + \phi \left( {\kappa_{f} - \kappa_{s} } \right)}}} \right]$

$\phi$ is the nano solid-particle size coefficient. $\mu_{f}$, $\rho_{f}$, $(C_{p} )_{f}$ and $\kappa_{f}$ are dynamical viscidness, intensity, functioning thermal capacity, and thermal conductivity of the standard fluid, respectively. The additional characteristics $\rho_{s}$, $(C_{p} )_{s}$ and $\kappa_{s}$ are the concentration, effective heat capacitance, and heat conductance of the nano molecules, correspondingly.

### Thermo-physical properties of P-EHNF

The primary assumption of hybrid nanofluids is the suspension of two distinct forms of nano solid particles inside the basis fluid^70^. This assumption improves the capacity for heat transmission of common liquids and is a higher heat interpreter than nanofluids. P-EHNF variables content is summarised in Table 2^71,72^.

**Table 2:** Thermo-physical features of hybrid nanofluids.

Features	Hybrid nanofluid
Viscidness $\left( \mu \right)$	$\mu_{hnf} = \mu_{f} (1 - \phi_{ST} )^{ - 2.5} (1 - \phi_{MT} )^{ - 2.5}$
Density $\left( \rho \right)$	$\rho_{hnf} = [(1 - \phi_{MT} )\{ (1 - \phi_{ST} )\rho_{f} + \phi_{ST} \rho_{{p_{1} }} \} ] + \phi_{Go} \rho_{{p_{2} }}$
Heat capacity $\left( {\rho C_{p} } \right)$	$(\rho C_{p} )_{hnf} = [\left( {1 - \phi_{MT} } \right)\{ \left( {1 - \phi_{ST} } \right)(\rho C_{p} )_{f} + \phi_{ST} (\rho C_{p} )_{{p_{1} }} \} ] + \phi_{MT} (\rho C_{p} )_{{p_{2} }}$
Thermal conductivity $\left( \kappa \right)$	$\frac{{\kappa_{hnf} }}{{\kappa_{gf} }} = \left[ {\frac{{\left( {\kappa_{{p_{2} }} + \left( {m - 1} \right)\kappa_{gf} } \right) - \left( {m - 1} \right)\phi_{ST} \left( {\kappa_{gf} - \kappa_{{p_{2} }} } \right)}}{{\left( {\kappa_{{p_{2} }} + \left( {m - 1} \right)\kappa_{gf} } \right) + \phi_{MT} \left( {\kappa_{gf} - \kappa_{{p_{2} }} } \right)}}} \right]$; $\frac{{\kappa_{gf} }}{{\kappa_{f} }} = \left[ {\frac{{\left( {\kappa_{{p_{1} }} + \left( {m - 1} \right)\kappa_{f} } \right) - \left( {m - 1} \right)\phi_{ST} \left( {\kappa_{f} - \kappa_{{p_{1} }} } \right)}}{{\left( {\kappa_{{p_{1} }} + \left( {m - 1} \right)\kappa_{f} } \right) + \phi_{ST} \kappa_{f} - \kappa_{{p_{1} }} )}}} \right]$

In Table 2, $\mu_{hnf}$, $\rho_{hnf}$, $\rho (C_{p} )_{hnf}$ and $\kappa_{hnf}$ are mixture nanofluid functional viscidness, concentration, exact thermal capacitance, and thermal conductance. $\phi$ is the volume of solid nano molecules coefficient for mono nanofluid and $\phi_{hnf} = \phi_{ST} + \phi_{MT}$ is the nano solid particles magnitude measurement for the combination nanofluid. $\mu_{f}$, $\rho_{f}$, $(C_{p} )_{f}$, $\kappa_{f}$ and $\sigma_{f}$ are functional viscidness, density, exact thermal capacity, and heat conductivity of the base fluid. $\rho_{{p_{1} }}$, $\rho_{{p_{2} }}$, $(C_{p} )_{{p_{1} }}$, $(C_{p} )_{{p_{2} }}$, $\kappa_{{p_{1} }}$ and $\kappa_{{p_{2} }}$ are the density, specific heat capacity, and thermal conductivity of the nano-molecules.

### Nano solid-particle shape-factor *m*

The scale of the multiple nano solid-particles is defined as the shaped-nanoparticles factor. Table 3 shows the importance of the experiential form factor for different particle forms (for instance, see Xu and Chen^73^).

**Table 3:** Shape-factor worth for different molecules shape.

Nanoparticles type	Shape	Size ($m$)	Sphericity
Sphere		3	1.0
Hexahedron		3.7221	0.87
Tetrahedron		4.0613	0.82
Column		6.3698	0.61
Lamina		16.1576	0.33

### Nano solid-particles and basefluid lineaments

In this analysis, the material characteristics of the primary oil-based liquid of the engine are specified in Table 4^74,75^.

**Table 4:** Fabricated materials thermo-physical attributes.

Thermophysical	$\rho$(kg m^−3^)	$c_{p}$(J kg^−1 ^K^−1^)	$k$(W mK^−1^)
SWCNTs	2600	425	6000
MWCNTs	1600	796	3000
Engine Oil (*EO*)	884	1910	0.144

### Rosseland approximation

Radiative flow only passes a shortened distance because its non-Newtonian P-EHNF is thicker. Because of this, the approximation for radiative fluxing from Rosseland^76^ is utilised in formula (4).

7 $$ q_{r} = - \frac{{4\sigma^{*} }}{{3k^{*} }}\frac{{\partial \yen^{4} }}{\partial y}, $$

herein, $\sigma^{*}$ signifies the constant worth of Stefan–Boltzmann and $k^{*}$ symbols the rate.

## Dimensionless formulations model

Given the similarity technology that transforms the governing PDEs into ODEs, the BVP formulas (2)–(6) are modified. Familiarising stream function $\psi$ in the formula^75^

8 $$ B_{1} = \frac{\partial \psi }{{\partial y}}, B_{2} = - \frac{\partial \psi }{{\partial x}}. $$

The specified similarity quantities are

9 $$ \Omega \left( {x,y} \right) = \sqrt {\frac{b}{{\nu_{f} }}} y,\quad \psi \left( {x,y} \right) = \sqrt {\nu_{f} b} xf\left( \Omega \right),\quad \theta \left( \Omega \right) = \frac{{\yen - \yen_{\infty } }}{{\yen_{w} - \yen_{\infty } }}. $$

into Eqs. (2)–(4). We get

10 $$   A_{1}^{*} f^{{'''}} \left( {1 - A_{2}^{*} f^{{''2}} } \right) + \phi _{b} \left[ {ff^{{''}}  - f^{{'2}} } \right] - \frac{1}{{\phi _{a} }}K_{\varsigma } f^{'}  = 0,   $$

11 $$ \theta ^{\prime\prime}\left( {1 + \frac{1}{{\phi_{d} }}P_{\varsigma } N_{\varsigma } } \right) + P_{\varsigma } \frac{{\phi_{c} }}{{\phi_{d} }}\left[ {f\theta ^{\prime} - f^{\prime}\theta + \frac{{E_{\varsigma } }}{{\phi_{a} \phi_{c} }}f^{{\prime\prime}{2}} - \varepsilon_{\varsigma } \left( {ff^{\prime}\theta ^{\prime} + f^{2} \theta ^{\prime\prime}} \right)} \right] = 0. $$

with

12 $$ \left. {\begin{array}{*{20}l} {f\left( 0 \right) = S, \,f^{\prime}\left( 0 \right) = 1 + {\Lambda }_{\varsigma } f^{\prime\prime}\left( 0 \right),\quad \theta ^{\prime}\left( 0 \right) = - H_{\varsigma } \left( {1 - \theta \left( 0 \right)} \right)} \hfill \\ {f^{\prime}\left( \Omega \right) \to 0, \theta \left( \Omega \right) \to 0,\quad as\quad \Omega \to \infty .} \hfill \\ \end{array} } \right\} $$

where $\phi ^{\prime}_{i} s$ is $a \le i \le d$ in formulas (10) and (11) signify the subsequent thermo-physical structures for P-HNF

13 $$ \phi_{a} = (1 - \phi_{ST} )^{2.5} (1 - \phi_{MT} )^{2.5} ,\quad \phi_{b} = \left( {1 - \phi_{MT} } \right)\left[ {\left( {1 - \phi_{ST} } \right) + \phi_{ST} \frac{{\rho_{{p_{1} }} }}{{\rho_{f} }}} \right] + \phi_{MT} \frac{{\rho_{{p_{2} }} }}{{\rho_{f} }}, $$

14 $$ \phi_{c} = \left( {1 - \phi_{MT} } \right)\left\{ {\left( {1 - \phi_{ST} } \right) + \phi_{ST} \frac{{(\rho C_{p} )_{{p_{1} }} }}{{(\rho C_{p} )_{f} }}} \right\} + \phi_{MT} \frac{{(\rho C_{p} )_{{p_{2} }} }}{{(\rho C_{p} )_{f} }}, $$

15 $$ \phi_{d} = \left[ {\frac{{\left( {\kappa_{{p_{2} }} + \left( {m - 1} \right)\kappa_{nf} } \right) - \left( {m - 1} \right)\phi_{MT} \left( {\kappa_{nf} - \kappa_{{p_{2} }} } \right)}}{{\left( {\kappa_{{p_{2} }} + \left( {m - 1} \right)\kappa_{nf} } \right) + \phi_{MT} \left( {\kappa_{nf} - \kappa_{{p_{2} }} } \right)}}} \right]\left[ {\frac{{\left( {\kappa_{{p_{1} }} + \left( {m - 1} \right)\kappa_{f} } \right) + \phi_{ST} \left( {\kappa_{f} - \kappa_{{p_{1} }} } \right)}}{{\left( {\kappa_{{p_{1} }} + \left( {m - 1} \right)\kappa_{f} } \right) - \left( {m - 1} \right)\phi_{ST} \left( {\kappa_{f} - \kappa_{{p_{1} }} } \right)}}} \right]. $$

### Explanation of the entrenched control constraints

Equation (2) is accurately confirmed. Previously, the representation $\mathrm{^{\prime}}$ existed for demonstrating the derivatives regarding $\Omega$.

**Table:** 

Symboles	Name	Formule	Default value
$A_{1}^{*}$	Prandtl–Eyring parameter-I	$A_{1}^{*} = \frac{{A_{{\text{d}}} }}{{\mu_{fC} }}$	1.0
$A_{2}^{*}$	Prandtl–Eyring parameter-II	$A_{2}^{*} = \frac{{b^{*} x^{2} }}{{2C^{2} \nu_{f} }}$	0.4
$\varepsilon_{\varsigma }$	Relaxation time parameter	$\varepsilon_{\varsigma } = b\lambda_{0}$	0.2
$P_{\varsigma }$	Prandtl number	$P_{\varsigma }$ = $\frac{{\nu_{f} }}{{\alpha_{f} }}$	6450
$\phi$	Volume fraction		0.18
$K_{\varsigma }$	Porosity parameter	$K_{\varsigma } = \frac{{\nu_{f} }}{bk}$	0.2
$S$	Suction/injection parameter	$S = - V_{\varsigma } \sqrt {\frac{1}{{\nu_{f} { }b}}}$	0.4
$N_{\varsigma }$	Thermal radiation parameter	$N_{\varsigma } = \frac{16}{3}\frac{{\sigma^{*} \yen_{\infty }^{3} }}{{\kappa^{*} \nu_{f} (\rho C_{p} )_{f} }}$	0.3
$E_{\varsigma }$	Eckert number	$E_{\varsigma } = \frac{{U_{w}^{2} }}{{(C_{p} )_{f} \left( {\yen_{w} - \yen_{\infty } } \right)}}$	0.3
$H_{\varsigma }$	Biot number	$H_{\varsigma } = \frac{{h_{\varsigma } }}{{k_{\varsigma } }}\sqrt {\frac{{\nu_{f} }}{b}}$	0.3
$m$	Shape parameter (spherical)	$m$	3
${\Lambda }_{\varsigma }$	Velocity slip	${\Lambda }_{\varsigma } = \sqrt {\frac{b}{{\nu_{f} }}} N_{\varsigma }$	0.3

### Drag-force and Nusselt number

The drag-force $\left( {C_{f} } \right)$ combined with the Nusselt amount $\left( {Nu_{x} } \right)$ are the interesting physical amounts that controlled the flowing and specified as^66^

16 $$ C_{f} = \frac{{\tau_{w} }}{{\frac{1}{2}\rho_{f} U_{w}^{2} }},\quad Nu_{x} = \frac{{xq_{w} }}{{k_{f} \left( {\yen_{w} - \yen_{\infty } } \right)}} $$

where $\tau_{w}$ and $q_{w}$ determine as

17 $$ \tau_{w} = \left( {\frac{{A_{{\text{d}}} }}{C}\frac{{\partial B_{1} }}{\partial y} + \frac{{A_{{\text{d}}} }}{{6C^{3} }}\left( {\frac{{\partial B_{1} }}{\partial y}} \right)^{3} } \right)_{y = 0} ,\quad q_{w} = - k_{hnf} \left( {1 + \frac{16}{3}\frac{{\sigma^{*} \yen_{\infty }^{3} }}{{\kappa^{*} \nu_{f} (\rho C_{p} )_{f} }}} \right)\left( {\frac{\partial \yen}{{\partial y}}} \right)_{y = 0} $$

The dimensionless transmutations (9) are implemented to obtain

18 $$ C_{f} Re_{x}^{\frac{1}{2}} = A_{1}^{*} f^{\prime\prime}\left( 0 \right) - \frac{1}{3}A_{1}^{*} A_{2}^{*} \left( {f^{\prime\prime}\left( 0 \right)} \right)^{3} ,\quad Nu_{x} Re_{x}^{{ - \frac{1}{2}}} = - \frac{{k_{hnf} }}{{k_{f} }}\left( {1 + N_{\varsigma } } \right)\theta ^{\prime}\left( 0 \right), $$

where $Nu_{x}$ means Nusselt aggregate and $C_{f}$ states drag force constant. $Re_{x} = \frac{{u_{w} x}}{{\nu_{f} }}$ is local $Re$ built in the extended swiftness $u_{w} \left( x \right)$.

## Classical Keller box technique

**Figure 2:**
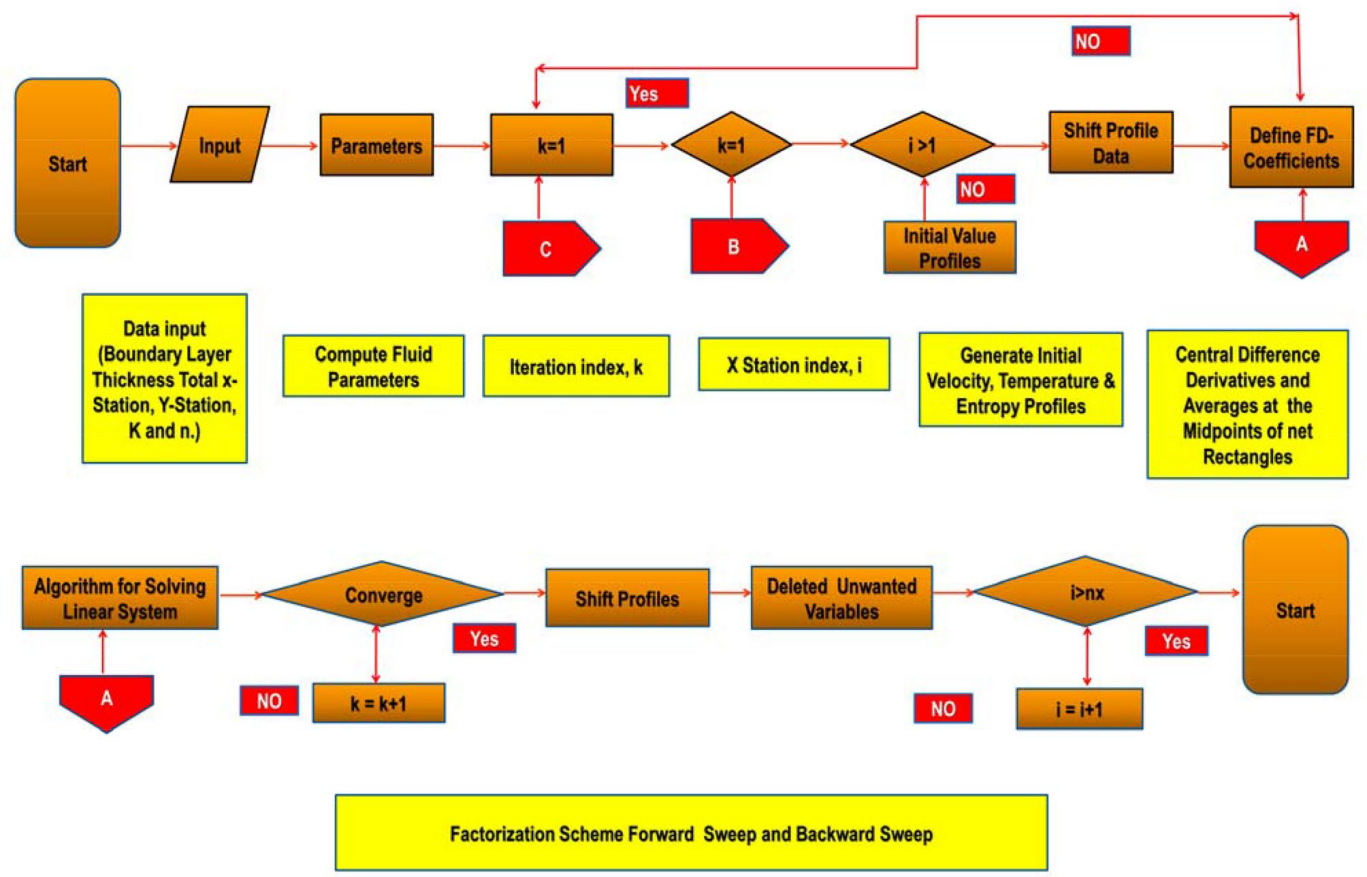
Chart of KBM steps.

Because of its rapid convergence, the Keller-box approach (KBM)^77^ is used to find solutions for model formulas (Fig. 2). KBM is used to find the localised solve of (10) and (11) with constraints (12). The policy of KBM is specified as next:

### Stage 1: ODEs adaptation

In the early stage, all of the ODEs must be changed into 1st-order ODEs (10)–(12)

19 $$ z_{1} = f^{\prime}, $$

20 $$ z_{2} = z_{1}^{^{\prime}} , $$

21 $$ z_{3} = \theta^{\prime}, $$

22 $$ A_{1}^{*} z_{2}^{^{\prime}} \left( {1 - A_{2}^{*} z_{2}^{2} } \right) + \phi_{b} \left[ {fz_{2} - z_{1}^{2} } \right] - \frac{1}{{\phi_{a} }}K_{\varsigma } z_{1} = 0, $$

23 $$ z_{3}^{^{\prime}} \left( {1 + \frac{1}{{\phi_{d} }}P_{\varsigma } N_{\varsigma } } \right) + P_{\varsigma } \frac{{\phi_{c} }}{{\phi_{d} }}\left[ {fz_{3} - z_{1} \theta + \frac{{E_{\varsigma } }}{{\phi_{a} \phi_{c} }}z_{2}^{2} - \varepsilon_{\varsigma } \left( {fz_{1} z_{3} + f^{2} z_{3}^{^{\prime}} } \right)} \right] = 0. $$

24 $$ f\left( 0 \right) = S,z_{1} \left( 0 \right) = 1 + {\Lambda }_{\varsigma } z_{2} \left( 0 \right),z_{3} \left( 0 \right) = - H_{\varsigma } \left( {1 - \theta \left( 0 \right)} \right),z_{1} \left( \infty \right) \to 0,\theta \left( \infty \right) \to 0. $$

### Stage 2: separation of domains

**Figure 3:**
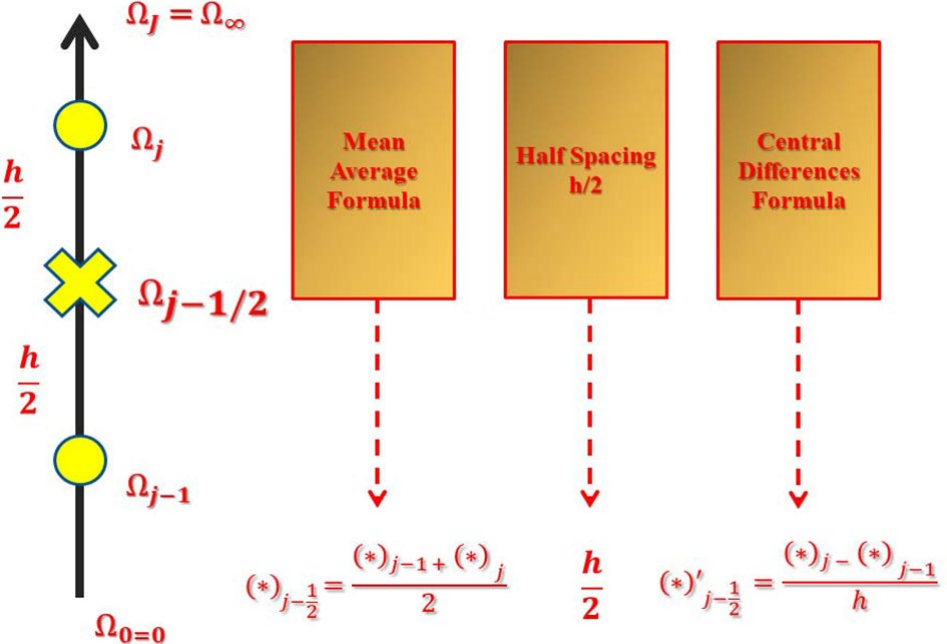
Net rectangle for showing difference approximations.

Discretisation plays a very important in the field of awareness. Discretising is usually conducted by making the area separated into equivalent-sized grids. Relatively lesser grids results are chosen in obtaining a higher precision for the calculation outcomes.

$$ \Omega_{0} = 0,\quad \Omega_{j} = \Omega_{j - 1} + h,\quad j = 1,2,3, \ldots ,J - 1,\quad \Omega_{J} = \Omega_{\infty } . $$

where $j$ is used for the spacing in $h$ in a horizontal direction to show the position of the coordinates. The solution to the problem is to be found without any initial approximation. It is very crucial for finding velocity, temperatures, temperature variations, and entropy to make a preliminary assumption between $\Omega = 0$ and $\Omega = \infty$. The frameworks from the result have been approximated solutions provided as they can happen the boundary conditions of the problem. It is imperative to remark that the results must be equalled with different preliminary estimations are chosen, but the replication computation and time are varied which have been taken for conducting the calculations (see Fig. 3):

By implementing significant differences, difference equivalences are figured, and functions are used to replace the mean values. The 1st-order ODEs (19)–(23) have been modified to algebraic formulas which are non-linear.

25 $$ \frac{{(z_{1} )_{j} + (z_{1} )_{j - 1} }}{2} = \frac{{f_{j} - f_{j - 1} }}{h}, $$

26 $$ \frac{{(z_{2} )_{j} + (z_{2} )_{j - 1} }}{2} = \frac{{(z_{1} )_{j} - (z_{1} )_{j - 1} }}{h}, $$

27 $$ \frac{{(z_{3} )_{j} + (z_{3} )_{j - 1} }}{2} = \frac{{\theta_{j} - \theta_{j - 1} }}{h}, $$

28 $$ \begin{aligned} & A_{1}^{*} \left( {\frac{{(z_{2} )_{j} - (z_{2} )_{j - 1} }}{h}} \right)\left( {1 - A_{2}^{*} \left( {\frac{{(z_{2} )_{j} + (z_{2} )_{j - 1} }}{2}} \right)^{2} } \right) \\ & \quad + \left[ {\phi_{b} \left( {\left( {\frac{{f_{j} + f_{j - 1} }}{2}} \right)\left( {\frac{{(z_{2} )_{j} + (z_{2} )_{j - 1} }}{2}} \right) - \left( {\frac{{(z_{1} )_{j} + (z_{1} )_{j - 1} }}{2}} \right)^{2} } \right) - K_{\varsigma } \frac{1}{{\phi_{a} }}\left( {\frac{{(z_{1} )_{j} + (z_{1} )_{j - 1} }}{2}} \right)} \right], \\ \end{aligned} $$

29 $$ \begin{aligned} & \left( {\frac{{(z_{3} )_{j} - (z_{3} )_{j - 1} }}{h}} \right)\left( {1 + \frac{1}{{\phi_{d} }}P_{\varsigma } N_{\varsigma } } \right) + P_{\varsigma } \frac{{\phi_{c} }}{{\phi_{d} }}\left[ {\left( {\frac{{f_{j} + f_{j - 1} }}{2}} \right)\left( {\frac{{(z_{3} )_{j} + (z_{3} )_{j - 1} }}{2}} \right)} \right] \\ & \quad + Pr\frac{{\phi_{c} }}{{\phi_{d} }}\left[ {\frac{{E_{\varsigma } }}{{\phi_{a} \phi_{c} }}\left( {\frac{{(z_{2} )_{j} + (z_{2} )_{j - 1} }}{2}} \right)^{2} - \left( {\frac{{(z_{1} )_{j} + (z_{1} )_{j - 1} }}{2}} \right)\left( {\frac{{\theta_{j} + \theta_{j - 1} }}{2}} \right)} \right] \\ & \quad - Pr\frac{{\phi_{c} }}{{\phi_{d} }}\left[ {\varepsilon_{\varsigma } \left( {\left( {\frac{{f_{j} + f_{j - 1} }}{2}} \right)\left( {\frac{{(z_{1} )_{j} + (z_{1} )_{j - 1} }}{2}} \right)\left( {\frac{{(z_{3} )_{j} + (z_{3} )_{j - 1} }}{2}} \right) + \left( {\frac{{f_{j} + f_{j - 1} }}{2}} \right)^{2} \left( {\frac{{(z_{3} )_{j} - (z_{3} )_{j - 1} }}{h}} \right)} \right)} \right] = 0. \\ \end{aligned} $$

### Stage 3: linearisation based on Newton’s method

The resulting formulas have been completed linearly by using Newton’s process. $\left( {i + 1} \right)^{th}$ iterations can be found in the earlier equations

30 $$ ()_{j}^{{\left( {i + 1} \right)}} = ()_{j}^{\left( i \right)} + {\ddot{\uptheta }}()_{j}^{\left( i \right)} . $$

By replacing this in (25) to (29) and after overlooking the higher-elevated bounds of ${\ddot{\uptheta }}_{j}^{i}$ a linear tri-diagonal equation scheme has been resulting as follows:

31 $$ {\ddot{\uptheta }}f_{j} - {\ddot{\uptheta }}f_{j - 1} - \frac{1}{2}h({\ddot{\uptheta }}(z_{1} )_{j} + {\ddot{\uptheta }}(z_{1} )_{j - 1} ) = (r_{1} )_{{j - \frac{1}{2}}} , $$

32 $$ {\ddot{\uptheta }}(z_{1} )_{j} - {\ddot{\uptheta }}(z_{1} )_{j - 1} - \frac{1}{2}h({\ddot{\uptheta }}(z_{2} )_{j} + {\ddot{\uptheta }}(z_{2} )_{j - 1} ) = (r_{2} )_{{j - \frac{1}{2}}} , $$

33 $$ {\ddot{\uptheta }}\theta_{j} - {\ddot{\uptheta }}\theta_{j - 1} - \frac{1}{2}h({\ddot{\uptheta }}(z_{3} )_{j} + {\ddot{\uptheta }}(z_{3} )_{j - 1} ) = (r_{3} )_{{j - \frac{1}{2}}} , $$

34 $$ \begin{array}{*{20}l}    & {(a_{1} )_{j} {\ddot{\uptheta }}f_{j}  + (a_{2} )_{j} {\ddot{\uptheta }}f_{{j - 1}}  + (a_{3} )_{j} {\ddot{\uptheta }}z_{{1_{j} }}  + (a_{4} )_{j} {\ddot{\uptheta }}z_{{1_{{j - 1}} }}  + (a_{5} )_{j} {\ddot{\uptheta }}z_{{2_{j} }}  + (a_{6} )_{j} {\ddot{\uptheta }}z_{{2_{{j - 1}} }} } \hfill  \\    &\quad { +\, (a_{7} )_{j} {\ddot{\uptheta }}\theta _{j}  + (a_{8} )_{j} {\ddot{\uptheta }}\theta _{{j - 1}}  + (a_{9} )_{j} {\ddot{\uptheta }}(z_{3} )_{j}  + (a_{{10}} )_{j} {\ddot{\uptheta }}(z_{3} )_{{j - 1}}  = (r_{4} )_{{j - \frac{1}{2}}} ,} \hfill  \\   \end{array} $$

35 $$ \begin{array}{*{20}l}   & {(b_{1} )_{j} {\ddot{\uptheta }}f_{j}  + (b_{2} )_{j} {\ddot{\uptheta }}f_{{j - 1}}  + (b_{3} )_{j} {\ddot{\uptheta }}z_{{1_{j} }}  + (b_{4} )_{j} {\ddot{\uptheta }}z_{{1_{{j - 1}} }}  + (b_{5} )_{j} {\ddot{\uptheta }}z_{{2_{j} }}  + (b_{6} )_{j} {\ddot{\uptheta }}z_{{2_{{j - 1}} }} } \hfill  \\  &\quad  { + (b_{7} )_{j} {\ddot{\uptheta }}\theta _{j}  + (b_{8} )_{j} {\ddot{\uptheta }}\theta _{{j - 1}}  + (b_{9} )_{j} {\ddot{\uptheta }}(z_{3} )_{j}  + (b_{{10}} )_{j} {\ddot{\uptheta }}(z_{3} )_{{j - 1}}  = (r_{5} )_{{j - \frac{1}{2}}} .} \hfill  \\   \end{array} $$

where

36 $$ (r_{1} )_{{j - \frac{1}{2}}} = - f_{j} + f_{j - 1} + \frac{h}{2}(z_{1} )_{j} + ((z_{1} )_{j - 1} ), $$

37 $$ (r_{2} )_{{j - \frac{1}{2}}} = - (z_{1} )_{j} + (z_{1} )_{j - 1} + \frac{h}{2}((z_{2} )_{j} + (z_{2} )_{j - 1} ), $$

38 $$ (r_{3} )_{{j - \frac{1}{2}}} = - \theta_{j} + \theta_{j - 1} + \frac{h}{2}((z_{3} )_{j} + (z_{3} )_{j - 1} ), $$

39 $$ \begin{aligned} (r_{4} )_{{j - \frac{1}{2}}} & = - h\left[ {A_{1}^{*} \left( {\frac{{(z_{2} )_{j} - (z_{2} )_{j - 1} }}{h}} \right)\left( {1 - A_{2}^{*} \left( {\frac{{(z_{2} )_{j} + (z_{2} )_{j - 1} }}{2}} \right)^{2} } \right)} \right] \\ & \quad - h\left[ {\left[ {\phi_{b} \left( {\left( {\frac{{f_{j} + f_{j - 1} }}{2}} \right)\left( {\frac{{(z_{2} )_{j} + (z_{2} )_{j - 1} }}{2}} \right) - \left( {\frac{{(z_{1} )_{j} + (z_{1} )_{j - 1} }}{2}} \right)^{2} } \right) - K_{\varsigma } \frac{1}{{\phi_{a} }}\left( {\frac{{(z_{1} )_{j} + (z_{1} )_{j - 1} }}{2}} \right)} \right]} \right], \\ \end{aligned} $$

40 $$  \begin{aligned}   (r_{5} )_{{j - \frac{1}{2}}}  &  =  - h\left[ {\left( {\frac{{((z_{3} )_{j}  - \left( {z_{3} )_{{j - 1}} } \right)}}{h}} \right)\left( {1 + \frac{1}{{\phi _{d} }}P_{\varsigma } N_{\varsigma } } \right) + \frac{{\phi _{c} Pr}}{{\phi _{d} }}\left( {\frac{{\left( {f_{j}  + f_{{j - 1}} } \right)((z_{3} )_{j}  + \left( {z_{3} )_{{j - 1}} } \right)}}{4}} \right)} \right] \\     & \quad  + h\frac{{\phi _{c} P_{\varsigma } }}{{\phi _{d} }}\left[ {\left( {\frac{{\left( {\theta _{j}  + \theta _{{j - 1}} } \right)\left( {z_{{1_{j} }}  + z_{{1_{{j - 1}} }} } \right)}}{4}} \right) - \frac{{E_{\varsigma } }}{{\phi _{1} \phi _{3} }}\left( {\frac{{(z_{2} )_{j}  + (z_{2} )_{{j - 1}} }}{2}} \right)^{2} } \right] \\     & \quad  + h\frac{{\phi _{c} P_{\varsigma } }}{{\phi _{d} }}\left[ {\varepsilon _{\varsigma } \left( {\left( {\frac{{f_{j}  + f_{{j - 1}} }}{2}} \right)\left( {\frac{{(z_{1} )_{j}  + (z_{1} )_{{j - 1}} }}{2}} \right)\left( {\frac{{(z_{3} )_{j}  + (z_{3} )_{{j - 1}} }}{2}} \right) - \left( {\frac{{f_{j}  + f_{{j - 1}} }}{2}} \right)^{2} \left( {\frac{{(z_{3} )_{j}  - (z_{3} )_{{j - 1}} }}{h}} \right)} \right)} \right]. \\  \end{aligned}   $$

Converted boundary conditions after the similarity process were given below

41 $$ {\ddot{\uptheta }}f_{0} = 0,{\ddot{\uptheta }}(z_{1} )_{0} = 0,{\ddot{\uptheta }}(z_{3} )_{0} = 0,{\ddot{\uptheta }}(z_{1} )_{J} = 0,{\ddot{\uptheta }}\theta_{J} = 0. $$

### Stage 4: the bulk scheme and eliminating

At the final, bulk tridiagonal matrix has been reached from the formulations in (30)–(35) as follows,

42 $$ {\text{F}}{\ddot{\uptheta }} = p, $$

where

43 $$ {\text{F}} = \left[ {\begin{array}{*{20}l} {A_{1} } \hfill & {C_{1} } \hfill & {} \hfill & {} \hfill & {} \hfill & {} \hfill \\ {B_{2} } \hfill & {A_{2} } \hfill & {C_{2} } \hfill & {} \hfill & {} \hfill & {} \hfill \\ {} \hfill & \ddots \hfill & \ddots \hfill & \ddots \hfill & {} \hfill & {} \hfill \\ {} \hfill & {} \hfill & \ddots \hfill & \ddots \hfill & \ddots \hfill & {} \hfill \\ {} \hfill & {} \hfill & {} \hfill & {B_{J - 1} } \hfill & {A_{J - 1} } \hfill & {C_{J - 1} } \hfill \\ {} \hfill & {} \hfill & {} \hfill & {} \hfill & {B_{J} } \hfill & {A_{J} } \hfill \\ \end{array} } \right],{\ddot{\uptheta }} = \left[ {\begin{array}{*{20}l} {{\ddot{\uptheta }}_{1} } \hfill \\ {{\ddot{\uptheta }}_{2} } \hfill \\ \vdots \hfill \\ {{\ddot{\uptheta }}_{j - 1} } \hfill \\ {{\ddot{\uptheta }}_{j} } \hfill \\ \end{array} } \right],p = \left[ {\begin{array}{*{20}l} {(r_{1} )_{{j - \frac{1}{2}}} } \hfill \\ {(r_{2} )_{{j - \frac{1}{2}}} } \hfill \\ \vdots \hfill \\ {(r_{J - 1} )_{{j - \frac{1}{2}}} } \hfill \\ {(r_{J} )_{{j - \frac{1}{2}}} } \hfill \\ \end{array} } \right]. $$

where $5 \times 5$ block-sized matrix is denoted by $F$ that corresponds to the size of $J \times J$. However, the vector of order $J \times 1$ is represented by ${\ddot{\uptheta }}$ and $p$. An esteemed LU factorising method is used for solving ${\ddot{\uptheta }}$ later. The equation $F{\ddot{\uptheta }} = p$ denotes that F with an array ${\ddot{\uptheta }}$ is used to yield a production array marked by $p$. Further, F is splinted into lower and upper trigonal matrices, i.e., $F = LU$ can be written as $LU{\ddot{\uptheta }} = p$. Let $U{\ddot{\uptheta }} = y$ tends to $Ly = p,$ which is used to provide the solution of $y$. Further, the values of $y$ computed are replaced into the equation $U{\ddot{\uptheta }} = y$ for solving ${\ddot{\uptheta }}$. The technique of back-substitution has been implemented as this is the easy method to find a solution.

## Code verification

On the other, by measuring the heat transmission rate outcomes from the current technique against the recent results available in the literature^78,79^, the validity of the method was evaluated. Table 5 summarises the comparing of reliabilities current during the researches. Nevertheless, the outcomes of the current examination are exceedingly accurate.

**Table 5:** Comparing of $- \theta^{\prime}\left( 0 \right)$ values with $P_{\varsigma }$, when $\phi = 0$, $\phi_{hnf} = 0$, $\varepsilon_{\varsigma } = 0$, ${\Lambda }_{\varsigma } = 0$, $E_{\varsigma } = 0$ $N_{\varsigma } = 0$, $S = 0$ and $H_{\varsigma } \to \infty$.

$Pr$	Ref.^78^	Ref.^79^	Present
72 × 10^−2^	080863135 × 10^−8^	080876122 × 10^−8^	080876181 × 10^−8^
1 × 10^0^	1 × 10^0^	1 × 10^0^	1 × 10^0^
3 × 10^0^	192,368,259 × 10^−8^	192,357,431 × 10^−8^	192,357,420 × 10^−8^
7 × 10^0^	307,225,021 × 10^−8^	307,314,679 × 10^−8^	307,314,651 × 10^−8^
10 × 10^0^	372,067,390 × 10^−8^	372,055,436 × 10^−8^	372,055,429 × 10^−8^

## Second law of thermodynamics

Porous media generally increase the entropy of the system. Jamshed et al.^80^ and Jamshed^81^ described the nanofluid entropy production by:

44 $$ E_{G} = \frac{{k_{hnf} }}{{\yen_{\infty }^{2} }}\left\{ {\left( {\frac{\partial \yen}{{\partial y}}} \right)^{2} + \frac{16}{3}\frac{{\sigma^{*} \yen_{\infty }^{3} }}{{\kappa^{*} \nu_{f} (\rho C_{p} )_{f} }}\left( {\frac{\partial \yen}{{\partial y}}} \right)^{2} } \right\} + \frac{{\mu_{hnf} }}{{\yen_{\infty } }}\left( {\frac{{\partial B_{1} }}{\partial y}} \right)^{2} + \frac{{\mu_{hnf} B_{1}^{2} }}{{k\yen_{\infty } }}. $$

The non-dimensional formulation of entropy analysis is as follows^82–84^,

45 $$ N_{G} = \frac{{\yen_{\infty }^{2} b^{2} E_{G} }}{{k_{f} \left( {\yen_{w} - \yen_{\infty } } \right)^{2} }}. $$

By formula (9), the non-dimensional entropy formula is:

46 $$ N_{G} = R_{e} \left[ {\phi_{d} \left( {1 + N_{\varsigma } } \right)\theta ^{{\prime}{2}} + \frac{1}{{\phi_{a} }}\frac{{B_{\varsigma } }}{\Pi }\left( {f^{{\prime\prime}{2}} + H_{\varsigma } f^{{\prime}{2}} } \right)} \right], $$

Here $R_{e}$ is the Reynolds number, $B_{\varsigma }$ signifies Brinkmann amount and $\Pi$ symbols the non-dimensional variation of the temperature.

## Results and discussion

**Figure 4:**
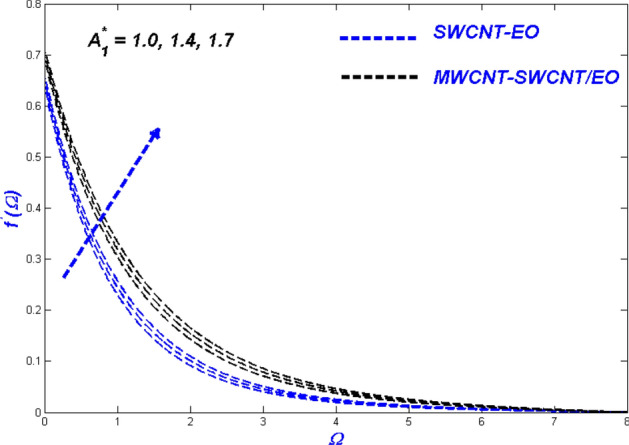
Velocity change with ${{A}_{1}}^{*}$.

**Figure 5:**
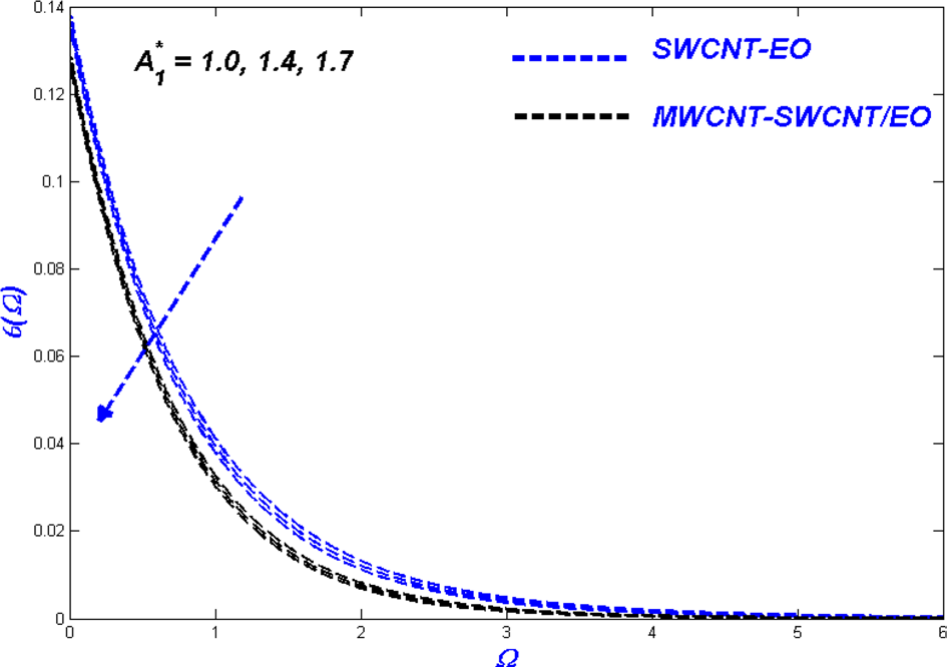
Temperature change with ${{A}_{1}}^{*}$.

**Figure 6:**
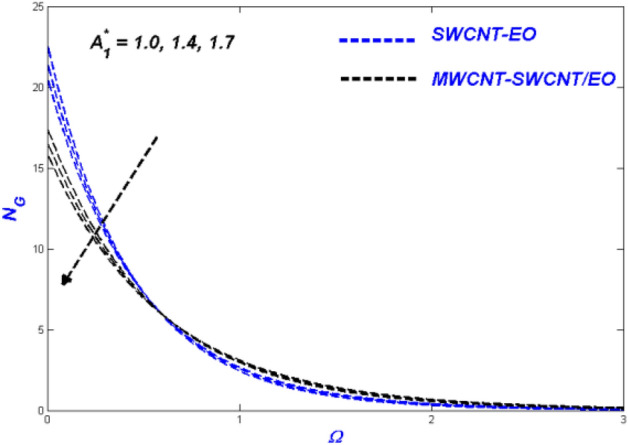
Entropy change with ${{A}_{1}}^{*}$.

**Figure 7:**
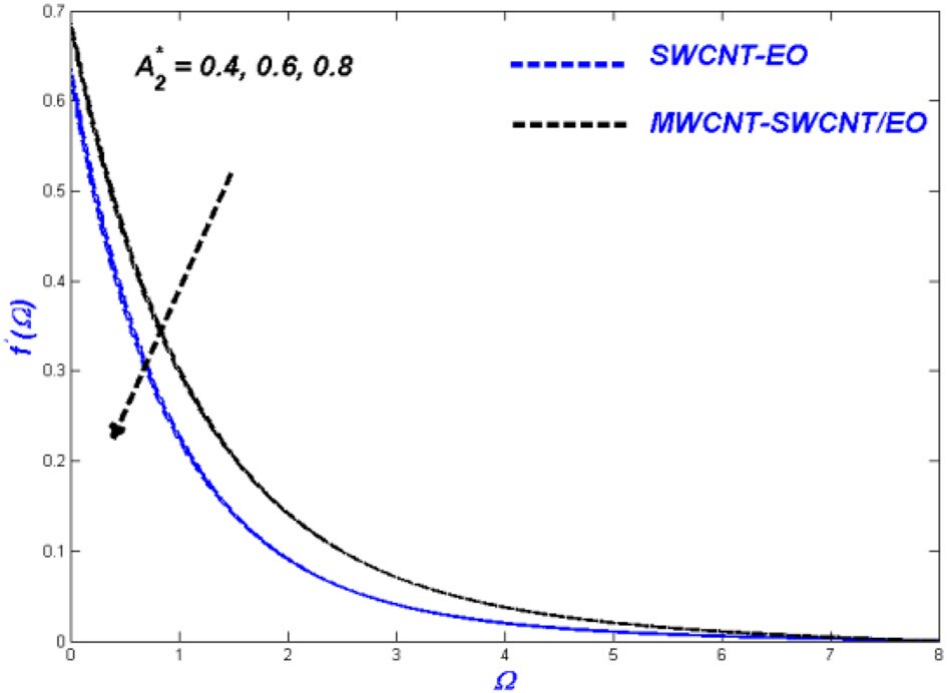
Velocity change with ${{A}_{2}}^{*}$.

**Figure 8:**
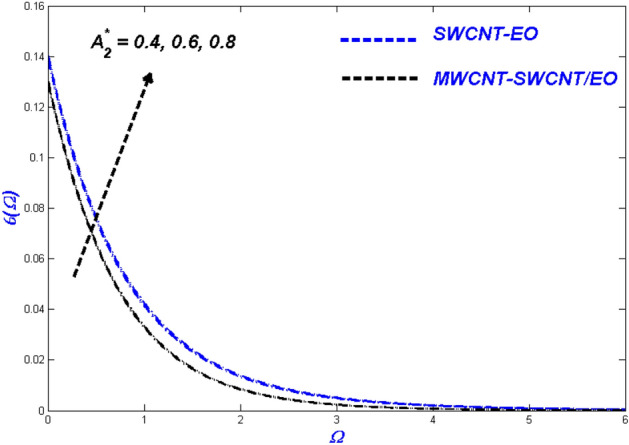
Temperature change with ${{A}_{2}}^{*}$.

**Figure 9:**
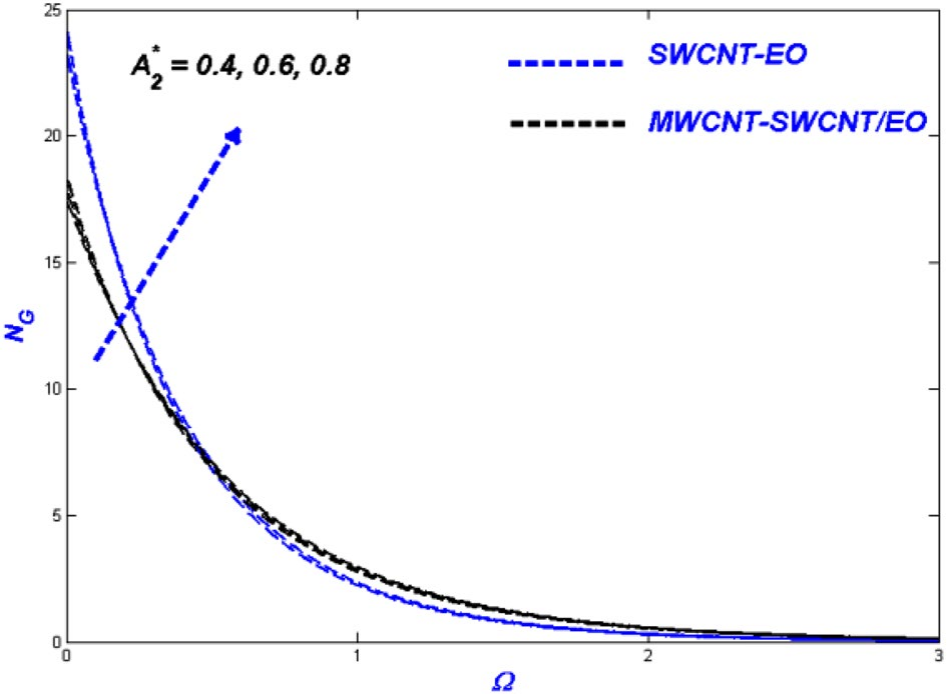
Entropy change with ${{A}_{2}}^{*}$.

**Figure 10:**
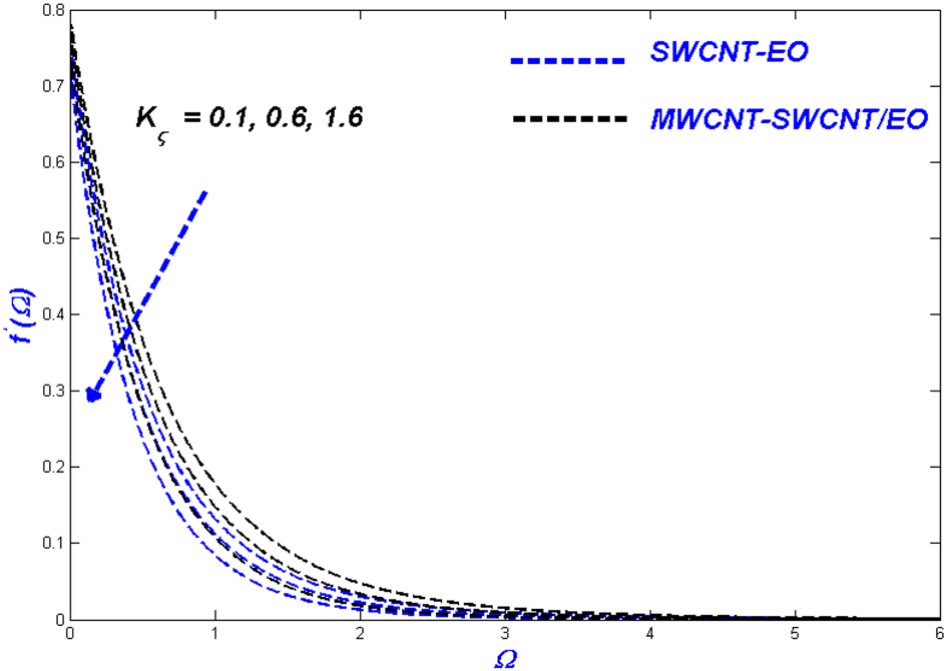
Velocity change with ${K}_{\varsigma }$.

**Figure 11:**
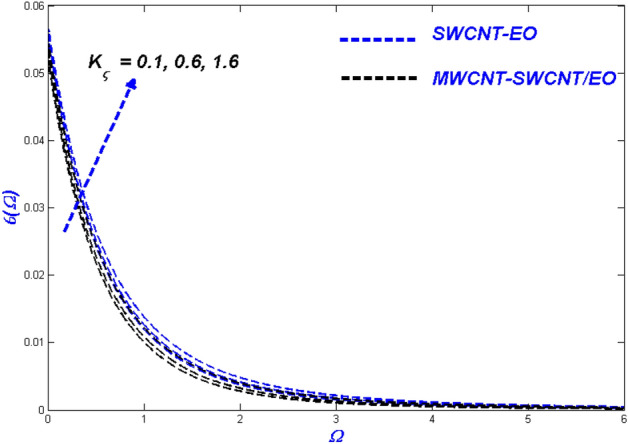
Temperature change with ${K}_{\varsigma }$.

**Figure 12:**
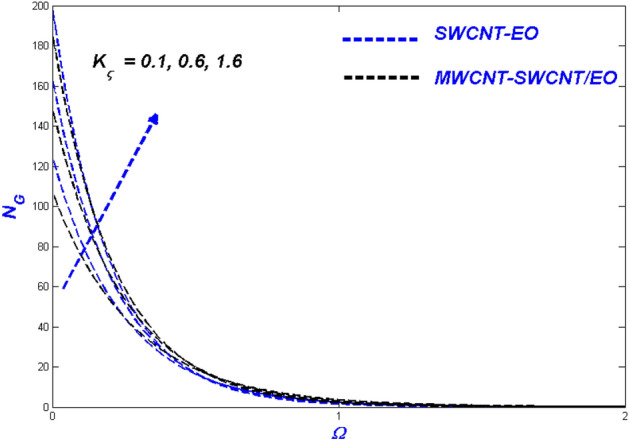
Entropy change with ${K}_{\varsigma }$.

**Figure 13:**
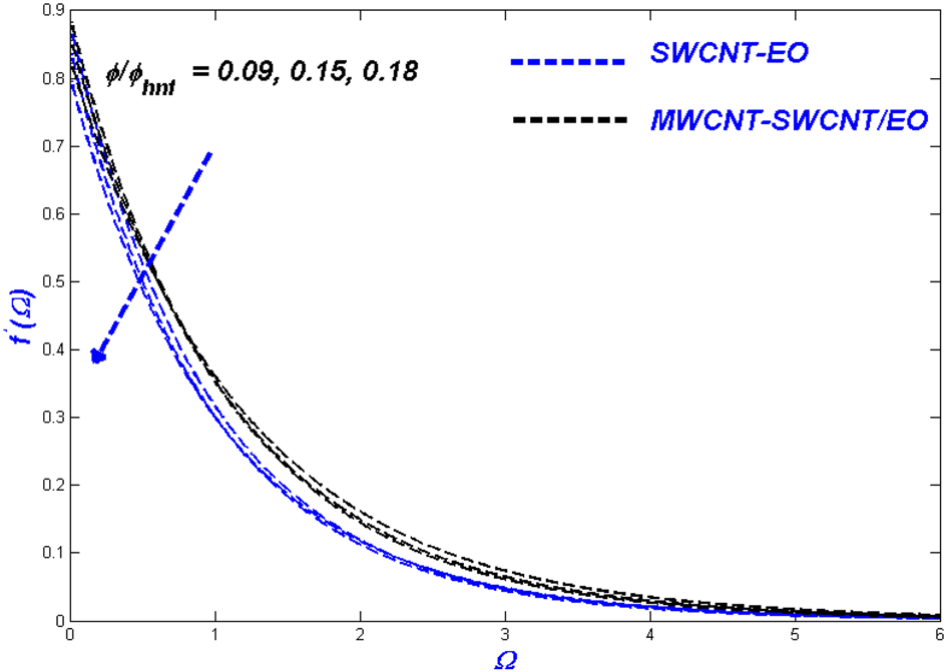
Velocity change with $\phi /{\phi }_{hnf}$.

**Figure 14:**
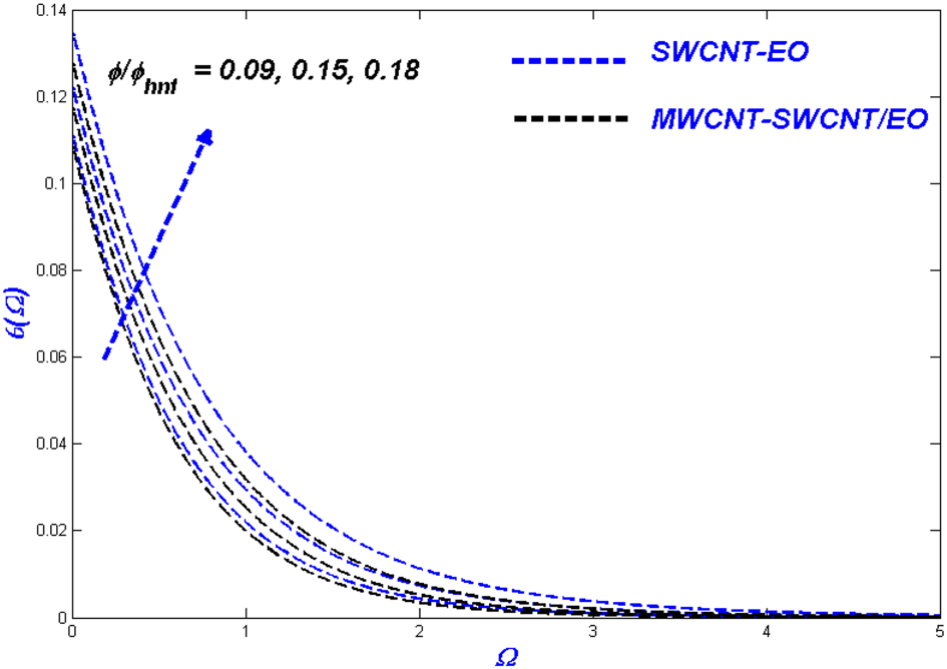
Temperature change with $\phi /{\phi }_{hnf}$.

**Figure 15:**
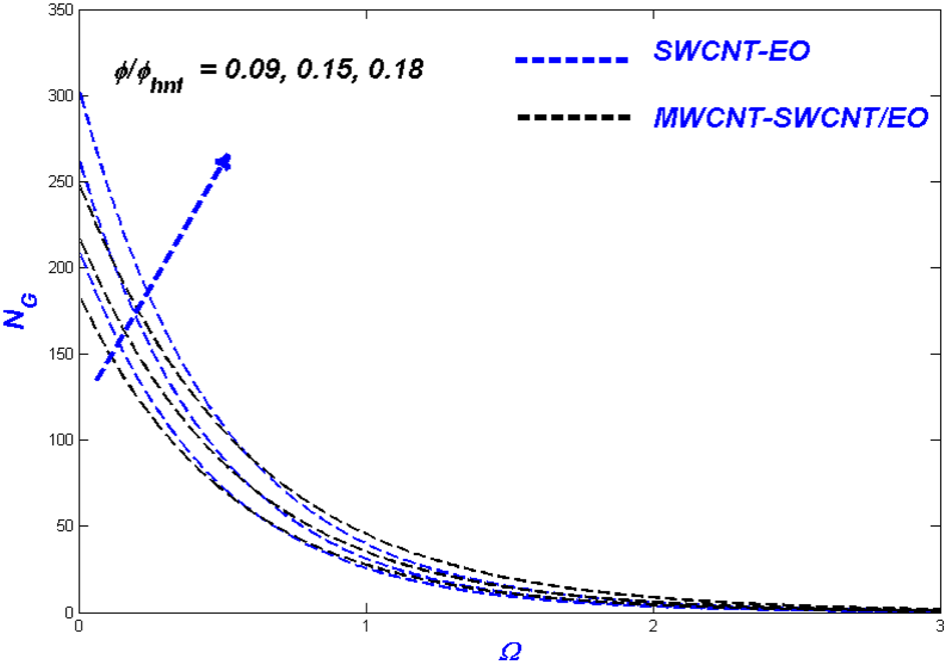
Entropy change with $\phi /{\phi }_{hnf}$.

**Figure 16:**
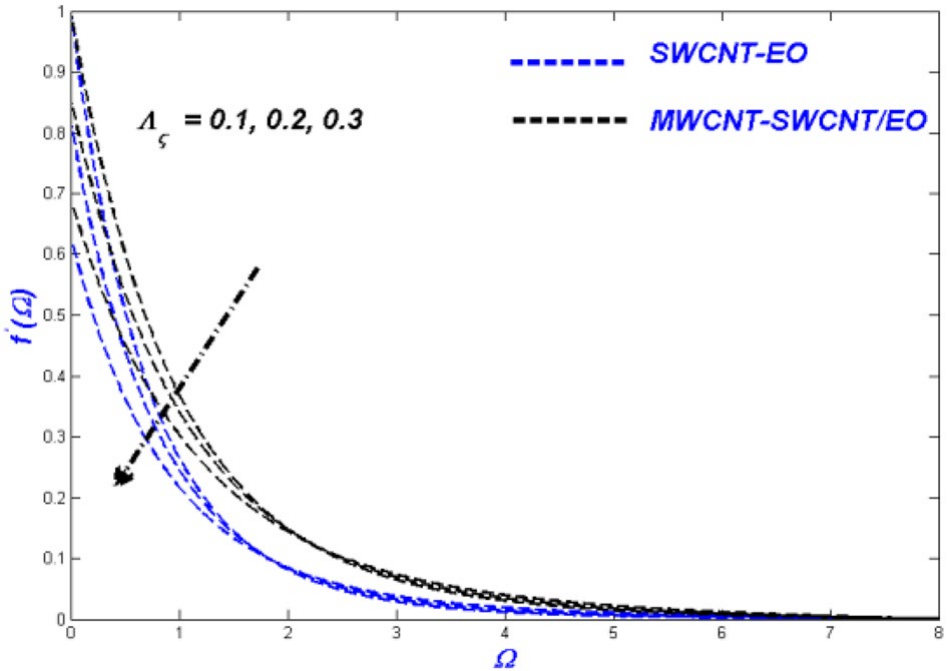
Velocity change with ${\Lambda }_{\varsigma }$.

**Figure 17:**
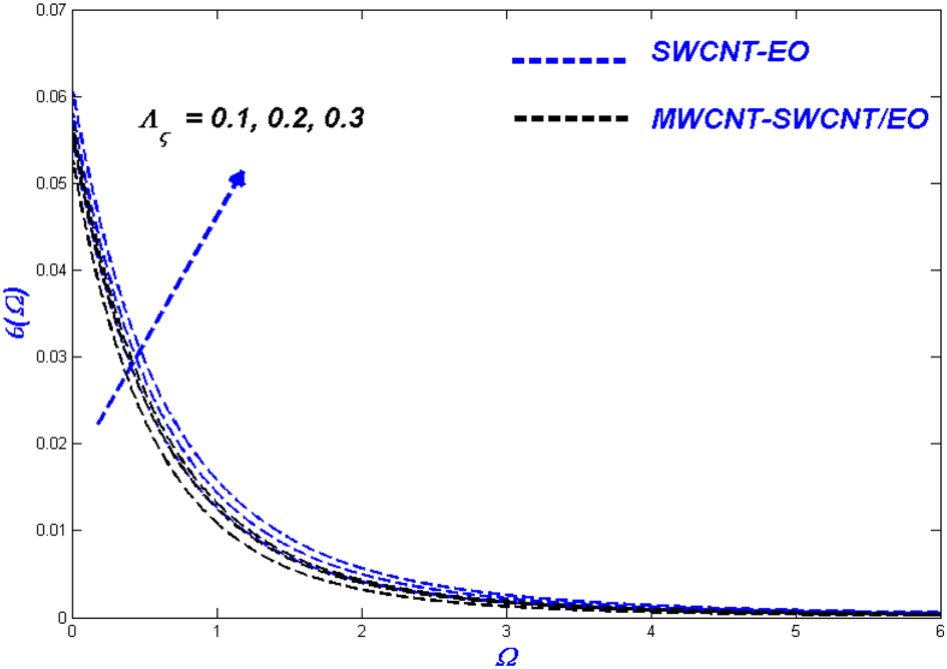
Temperature change with ${\Lambda }_{\varsigma }$.

**Figure 18:**
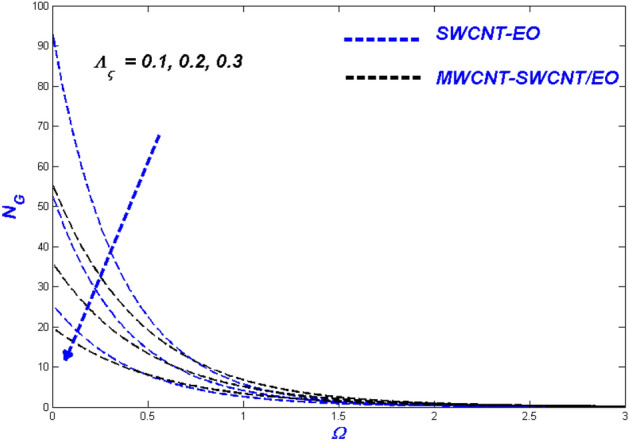
Entropy change with ${\Lambda }_{\varsigma }$.

**Figure 19:**
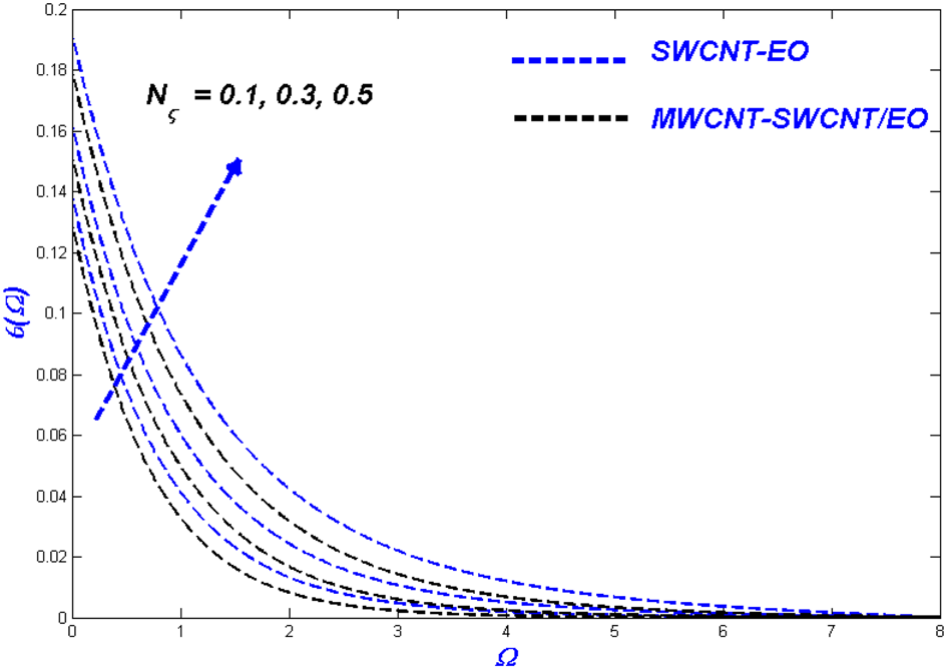
Temperature change with ${N}_{\varsigma }$.

**Figure 20:**
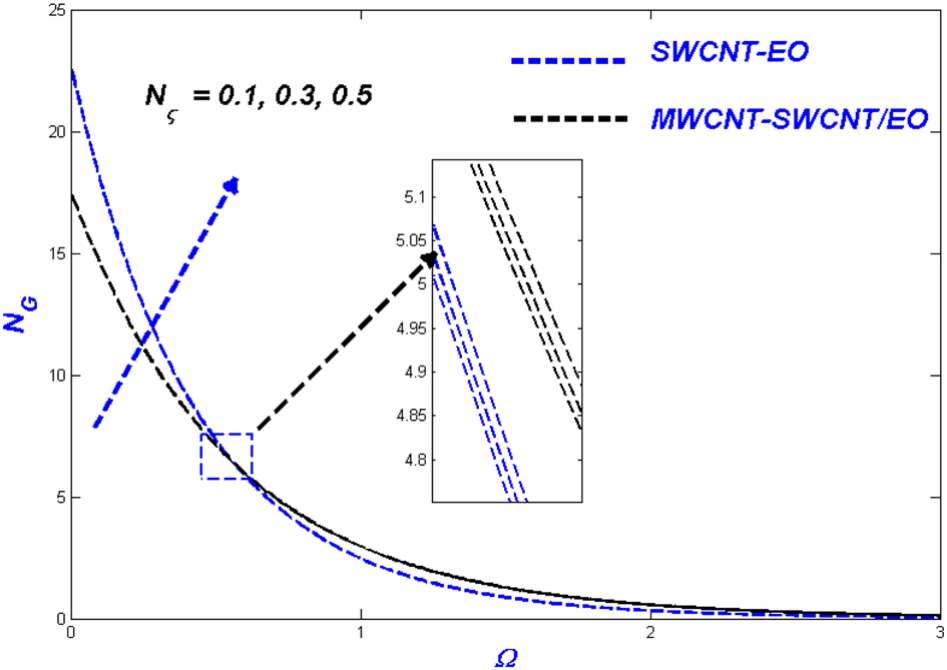
Entropy change with ${N}_{\varsigma }$.

**Figure 21:**
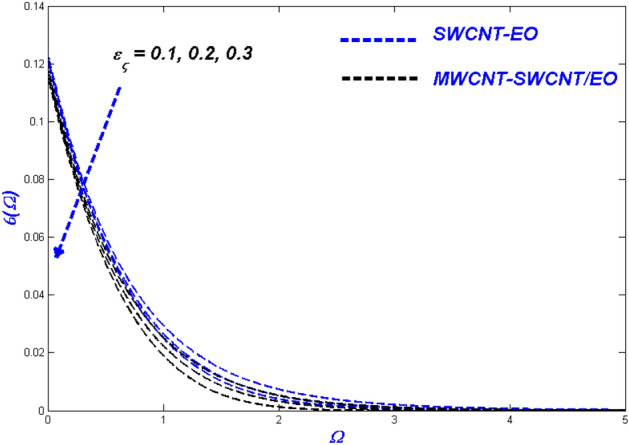
Temperature change with ${\varepsilon }_{\varsigma }$.

**Figure 22:**
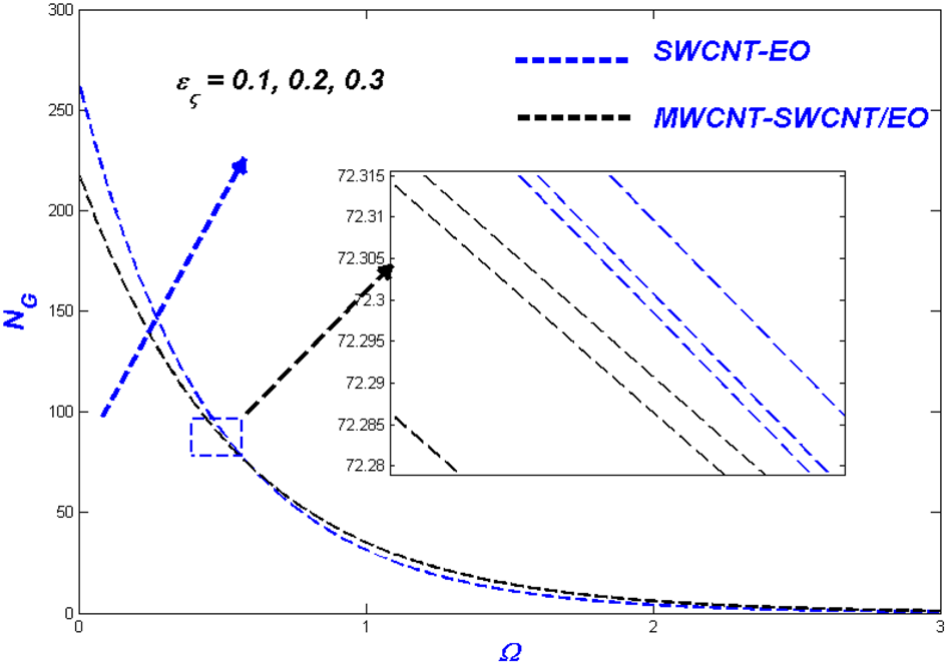
Entropy change with ${\varepsilon }_{\varsigma }$.

**Figure 23:**
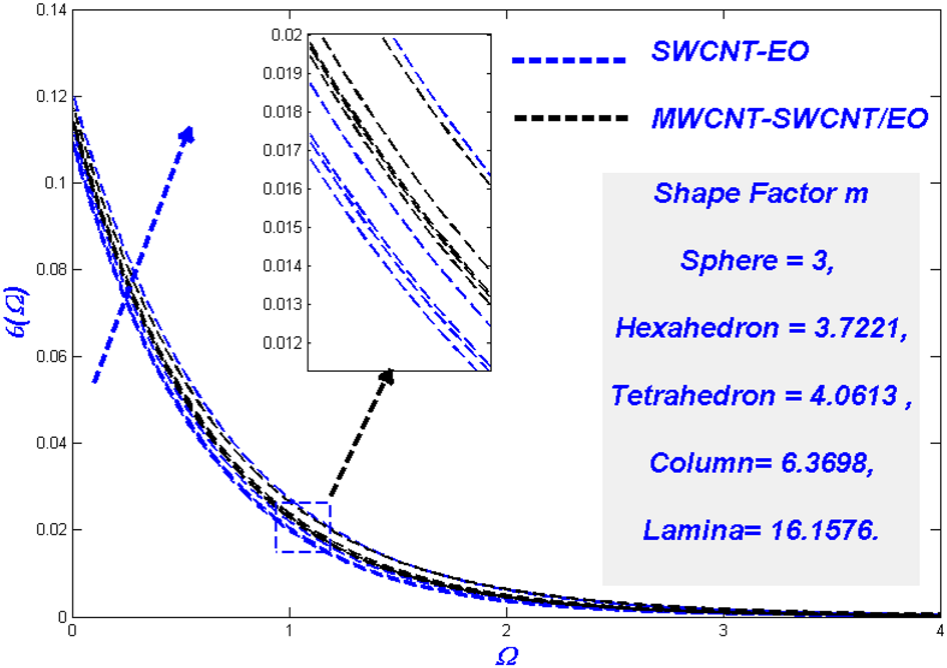
Temperature change with $m$.

**Figure 24:**
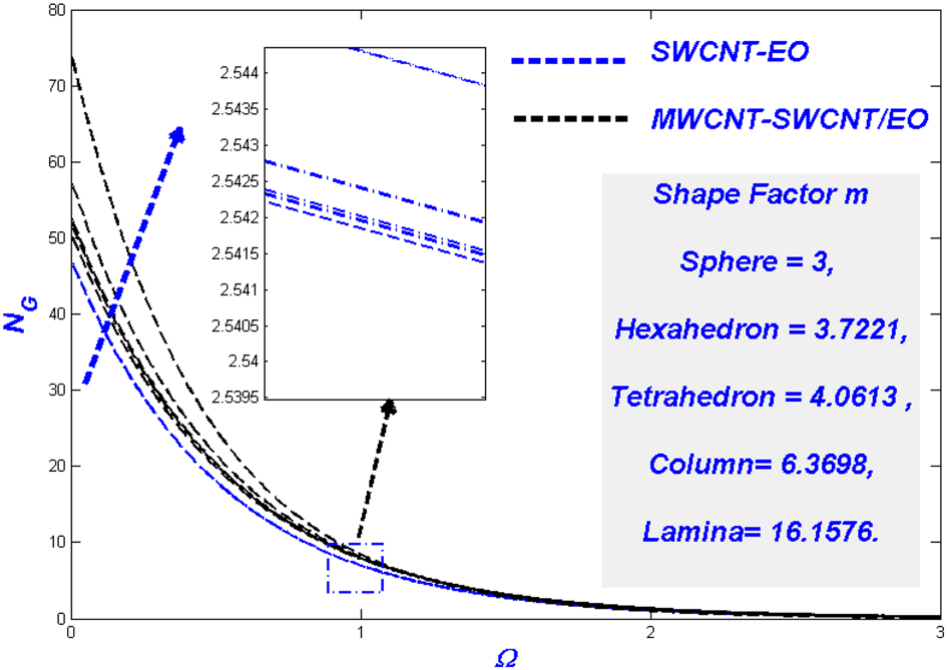
Entropy change with $m$.

**Figure 25:**
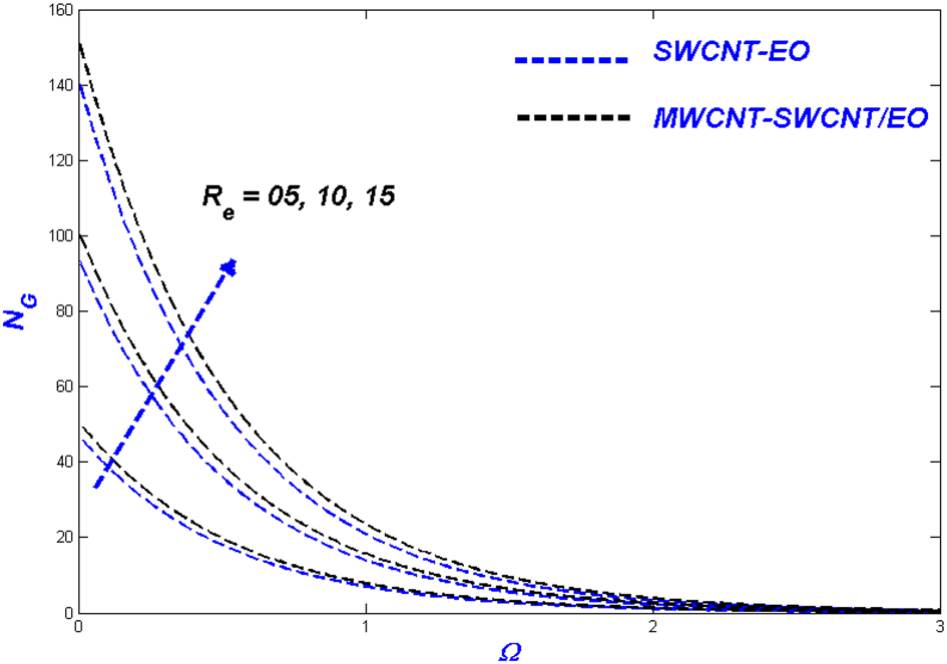
Entropy change with ${R}_{e}$.

**Figure 26:**
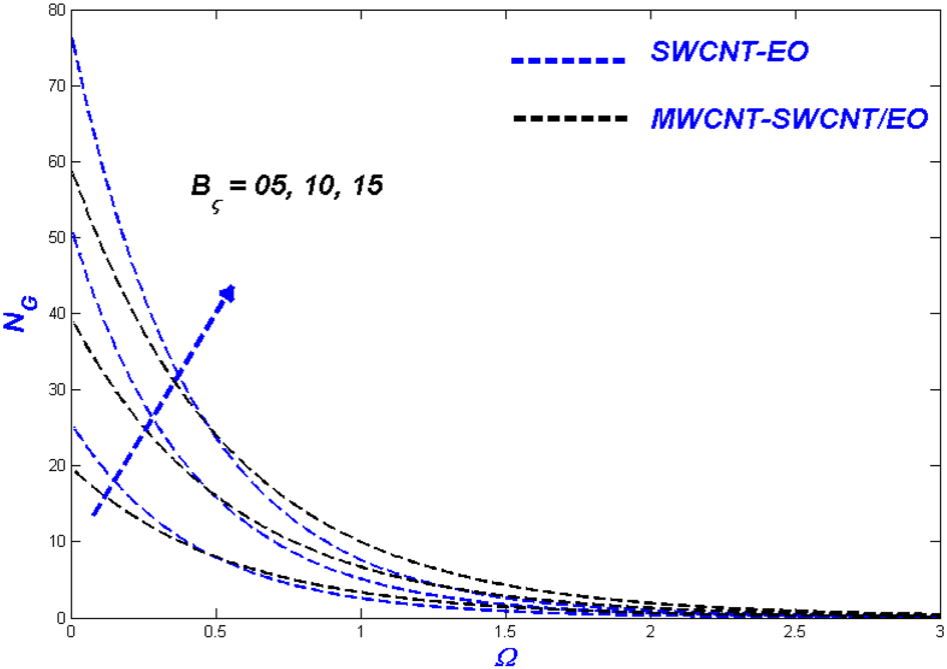
Entropy change with ${B}_{\varsigma }$.

An adequate discussion is indicated by numerical results that reach the model described before. As a result of these potential parameters, the values for $A_{1}^{*}$, $A_{2}^{*}$, $K_{\varsigma }$, $\phi$, ${\Lambda }_{\varsigma }$, $S$, $N_{\varsigma }$,${ }\varepsilon_{\varsigma }$, $E_{\varsigma }$ $H_{\varsigma } , R_{e}$ and $B_{\varsigma }$ been illustrated. These parameters show the physical performance of the non-dimensional quantities in Figs. 4, 5, 6, 7, 8, 9, 10, 11, 12, 13, 14, 15, 16, 17, 18, 19, 20, 21, 22, 23, 24, 25 and 26, such as velocity, energy, and entropy production. The results are obtained for Cu-EO normal P-ENF and MWCNT-SWCNT/EO non-Newtonian P-EHNF. The coefficient of skin friction and temperature variations are shown in Table 6. For example, the default values were 1.0 for $A_{1}^{*}$ and 0.4 for $A_{2}^{*}$, $K_{\varsigma }$ was set to be equal to 0.1, and $\phi$ = 0.18, $\phi_{MT}$ was set to 0.09, ${\Lambda }_{\varsigma }$ was set to 0.3, $S$ was set to 0.4, and $N_{\varsigma }$ was set to 0.3, $\varepsilon_{\varsigma }$ was set to 0.1, $E_{\varsigma }$ was set to 0.3, $H_{\varsigma }$ was set to 0.3, and $R_{e}$ and $B_{\varsigma }$ was set to 5.

**Table 6:** Values of ${C}_{f}R{e}_{x}^{1/2}$ and ${Nu}_{x}R{e}_{x}^{-1/2}$ for ${P}_{\varsigma }=6450$.

$A_{1}^{*}$	$A_{2}^{*}$	$K_{\varsigma }$	$\phi$	$\phi_{MT}$	${\Lambda }_{\varsigma }$	$S$	$N_{\varsigma }$	$\varepsilon_{\varsigma }$	$H_{\varsigma }$	$C_{f} Re_{x}^{\frac{1}{2}}$ SWCNT-EO	$C_{f} Re_{x}^{\frac{1}{2}}$ MWCNT-SWCNT/EO	$N_{u} Re_{x}^{{\frac{ - 1}{2}}}$ SWCNT-EO	$N_{u} Re_{x}^{{\frac{ - 1}{2}}}$ MWCNT-SWCNT/EO
1.0	0.4	0.1	0.18	0.09	0.3	0.4	0.3	0.2	0.3	4.7980	5.4521	2.5615	3.0496
1.4										4.8262	5.4884	2.5974	3.0727
1.7										4.8561	5.5173	2.6299	3.1092
	0.4									4.7980	5.4521	2.5615	3.0496
	0.6									4.7629	5.4264	2.5426	3.0138
	0.8									4.7487	5.3933	2.5273	3.0095
		0.1								4.7980	5.4521	2.5615	3.0496
		0.6								4.8113	5.4816	2.5381	3.0230
		1.6								4.8335	5.5250	2.5045	3.0045
			0.09							4.7372	–	2.5126	–
			0.15							4.7543	–	2.5458	–
			0.18							4.7980	–	2.5615	–
				0.0						–	4.7372	–	2.5126
				0.06						–	5.4377	–	3.0123
				0.09						–	5.4521	–	3.0496
					0.1					4.8626	5.5143	2.6083	3.0911
					0.2					4.8397	5.4916	2.5851	3.0648
					0.3					4.7980	5.4521	2.5615	3.0496
						0.2				4.7647	5.4305	2.5229	3.0092
						0.4				4.7980	5.4521	2.5615	3.0496
						0.6				4.8131	5.4845	2.5882	3.0675
							0.1			4.7980	5.4521	2.5237	3.0282
							0.3			4.7980	5.4521	2.5615	3.0496
							0.5			4.7980	5.4521	2.5925	3.0755
								0.1		4.7980	5.4521	2.5949	3.0754
								0.2		4.7980	5.4521	2.5615	3.0496
								0.3		4.7980	5.4521	2.5337	3.0042
									0.1	4.7980	5.4521	2.5359	3.0148
									0.3	4.7980	5.4521	2.5615	3.0496
									0.5	4.7980	5.4521	2.5979	3.0636

### Influence of Prandtl–Eyring parameter $A_{1}^{*}$

Figures 4, 5 and 6 illustrate the influence of the Prandtl–Eyring parameter $A_{1}^{*}$ on the velocity, energy, and entropy distributions of the Prandtl–Eyring hybrid nanofluid, respectively. $A_{1}^{*} {{^{\prime}s}}$ velocity fluctuation ($f^{\prime}$) is seen in Fig. 4. As the value of $A_{1}^{*}$ was elevated, so was the velocity profile for both fluids. The physical reason for this occurrence is that it causes the fluid's viscosity to decrease, reducing resistance while boosting fluid velocity. MWCNT-SWCNT nanofluid, on the other hand, has faster acceleration than SWCNT nanofluid. It can be explained as the hybrid nanofluid have an enormous density impact rather than the nanofluid. The temperature curve for the Prandtl–Eyring parameter $A_{1}^{*}$ is shown in Fig. 5. MWCNT-SWCNT hybrid nanofluid had a lower temperature profile since the value of $A_{1}^{*}$ was raised, while the Cu nanofluid had a higher temperature profile. More heat can be conveyed faster when the caused in this lowered manner due to velocity improve and expand. Another important distinction is that the hybrid nanofluid exhibits significantly reduced thermal conductivity when compared to pure nanofluid. Figure 6 depicted the Prandtl–Eyring hybrid nanofluid entropy fluctuation based on its parameter $A_{1}^{*}$. The quantity of entropy produced decreased as the amount of $A_{1}^{*}$ enhanced. MWCNT-SWCNT fluid exhibited a lower entropy value than SWCNT hybrid nanofluid, even though their values were the same at one point in the graph. This phenomenon occurs due to the low temperatures reducing hybrid nanofluid mobility, causing the system's entropy to proliferation.

### Influence of Prandtl–Eyring parameter $A_{2}^{*}$

There was an influence of Prandtl–Eyring Parameter $A_{2}^{*}$ on the Prandtl–Eyring hybrid nanofluid temperature, velocity, and entropy production profile (see Figs. 7, 8). Figure 7 depicts the varying $A_{2}^{*} {\text{ with velocity}}$. The velocity profile narrows as $A_{2}^{*}$ rises, with MWCNT-SWCNT/EO achieving a higher top speed than SWCNT-EO. Hybrid nanofluid particles have resistance due to the fact that they vary inversely with momentum diffusivity. As a result, the flow's velocity will be reduced with $A_{2}^{*}$. This phenomenon is because SWCNT-EO has a higher density and hence has a thicker flow than MWCNT-SWCNT/EO, making the fluid challenging to transport. Figure 8 shows the temperature change after $A_{2}^{*}$ has had its impact. As the value of $A_{2}^{*}$ grew, so did the temperature, with SWCNT-EO quickly reaching the desired temperature. The occurrence happens because the flow velocity dropped, and as a result, the heat transmission from the surface was degraded. Figure 9 shows the change in entropy according to the Prandtl–Eyring parameter $A_{2}^{*}$. The entropy profile grew as the value of $A_{2}^{*}$ grew, showing a clear connection between the two. It suggested that $A_{2}^{*}$ amplifying the impediment in the system, resulting in the entropy of the developing system being elevated.

### Effect of porous media variable $K_{\varsigma }$

Figures 10, 11 and 12 demonstrate that surface porosity affects several outputs, including flow speed, domain heat, and entropy generation. Improving the variable ($K_{\varsigma }$) in Fig. 10 makes the surface more porous, allowing more fluid to flow through it. Due to the other particles, the hybrid nanofluid moves more slowly through the porous surface when compared to MWCNT-SWCNT/EO Prandtl–Eyring nanofluid. This occurrence might be because the added particles delay the hybrid nanofluid's flow through the porous surface. Figure 11 displays the expansion of the porous medium variable ($K_{\varsigma }$) results in better heat dispersion throughout the domain. When a hole is made in a porous medium, the flow slows down, allowing more time to collect heat from the surface. This phenomenon improves the thermal distribution around the area. Since particle motions across porous media are sluggish, the porosity aids in the irreversibility of energy transfer across the domain during entropy production $\left( {N_{G} } \right)$ (Fig. 12).

### Effect of nanomolecules size $\phi$ and $\phi_{hnf}$

The efficacy of the nanofluid and hybrid versions appears to be determined by the fractional nanoparticle size in the base fluid. The more excellent fractional range of nanoparticles reduces flowability because of the additional load it adds. For some reason, the fractional upgrade prefers the hybrid nanofluid over the single-nanofluid, which flows lower in Fig. 13. This incidence displayed the primary reason for utilising nano- and hybrid-based fluid mixtures because of their exceptional heat transmission properties. This degradation occurs as a result of excessive nanoparticle surface area and higher hybrid nanofluid density. As the fractional volume of both kinds of flow fluids improved, so did the resultant thermal distribution, as shown in Fig. 14. Because of the temperature difference, when the nano molecule size is reduced, the molecules will disperse in the far-field flow. The thermal boundary layer's thickness will rise as a result of this change. The minimal size of nano molecules can be utilised to create the lowest possible temperature profile, as determined through experimentation. Figure 15 exhibits the leading nanofluid varies in the middle and settles down to the hybrid nanofluid at the far end, with energy entropy fluctuations also intensifying for fractional volume. SWCNT-EO has a greater entropy than MWCNT-SWCNT/EO because the hybrid nanofluid has a far higher thermal conductivity than nanofluid.

### Effect of velocity slip variable $\Lambda_{\varsigma }$

Figures 16, 17 and 18 evaluate the impact of enhanced slip circumstances on flow nature, thermal features, and entropy forms. Figure 16 illustrates the flow conditions in Prandtl–Eyring fluid mixtures are primarily centred upon the viscous behaviour. Due to this occurrence, slip conditions become incredibly critical in fluids as a whole. For a hybrid suspended Prandtl–Eyring nanofluid, the viscous nature and higher levels of flow slip generate more complex fluidity circumstances, with the result that the fluidity of the single nanofluid drops even more rapidly. Due to the flow hierarchy, the SWCNT-EO nanofluid maintains a higher temperature state than the MWCNT-SWCNT/EO hybrid nanofluid, which is depicted in Fig. 17. The improvement in boundary layer viscosity due to the decline in velocity will have a similar effect. As a result, it will have skyrocketed the flow's temperature. Because the hybrid nanofluid has less viscosity than the conventional nanofluid, it is predicted MWCNT-SWCNT/EO to have a lower temperature than SWCNT-EO. A descending trend in entropy formation can be seen for higher slip parameters because the slipped flow acts against the domain's entropy formation.

### Thermal radiative variable $N_{\varsigma }$ and relaxation time parameter ($\varepsilon_{\varsigma }$) influence

Figures 19 and 20 highlights the actual status of thermal diffusion and entropy generation under enhanced heat radiative flow limitation $\left( {N_{\varsigma } } \right).$ Thermally diffusing nanofluids have a propensity to rise in temperature past the interesting domain, boosting the heat transmission burden for radiation constrictions on the transient nanofluid. This temperature rise may be explained in a physical sense by supposing that thermal radiation is converted into electromagnetic energy. As a result, the distance from the surface from which radiation is emitted rises, ultimately superheating the boundary layer flow. As a result, the thermal radiative variable is critical in determining the system's temperature profile. A limit on radiative flow $\left( {N_{\varsigma } } \right)$ via entropy generation is illustrated in Fig. 20 by the overfilled dispersions. For different $\left( {N_{\varsigma } } \right)$ values, the entropic side-by-side leans toward developing more in MWCNT-SWCNT/EO than in SWCNT-EO nanofluid. A reasonable explanation for this occurrence is the system's irreparable heat transfer mechanism is entirely irreversible. According to Fig. 21, greater values of the relaxation time parameter cause a rise in the temperature of the Tangent hyperbolic hybrid nanofluids, as seen in the graph. As the temperature drops, the thickness of the thermal boundary layer reduces. Table 5 shows that when the rate of heat output efficiency, the effectiveness of the thermal system improves as well. Figure 22 shows the impact of engine oil-based nanofluid entropy profiles. The velocity profile, on the other hand, shows no change, while the entropy of the system increases with varying values $\varepsilon_{\varsigma }$.

### Effect of the diverse solid particle shape *m*

It is well-known that NPs have high thermal conductivity and transfer rates under a variety of physical conditions. In porous medium difficulties, such nano-level particles become an issue, modelled using the shape variable (m) in this study. From spherical (m = 3) to lamina (m = 16.176), the forms considered here ranged. To improve the thermal state, Fig. 23 indicates that nanoparticle shapes impact it. In comparison to SWCNT-EO mono nanofluid, the MWCNT-SWCNT/EO hybrid nanofluid has a more significant form impact. Hybrid nanofluid has a broader thermal layer boundary and a more excellent thermal distribution than nanofluid. Even in the MWCNT-SWCNT/EO hybrid nanofluid, the lamina (m = 16.176) shaped particles remain ahead of the others. The main physical reason for this phenomenon is the lamina shape particles has the most remarkable viscosity while the sphere has the minimum viscosity. It is also noted that at a higher temperature, the viscosity of the particles will be diminished. This phenomenon happens because of the temperature-dependent shear-thinning characteristic. The profiles in Fig. 24 indicate the form factors have a more substantial influence in MWCNT-SWCNT/EO NHF, which has a higher entropy rate than SWCNT-EO mono nanofluid, even though the morphologies of the particles have a much less impact.

### Entropy variations for Reynolds number ($R_{e}$) and Brinkman number ($B_{\varsigma }$)

Figure 25 depicted as Reynolds number proliferations $\left( {R_{e} } \right)$, the entropy rate $\left( {N_{G} } \right)$ improves as well. An aggregate Reynolds number supports nanoparticle mobility in porous media because of the dominance of inertial over viscous forces in the system. Consequently, entropy can be generated over the domain. MWCNT-SWCNT/EO HNF generated a higher entropy rate than MWCNT-SWCNT/EO nanofluid because of the combined efficiency of the particles. The Brinkman number $\left( {B_{\varsigma } } \right)$ was used to describe the heat created by viscous properties because it enhances the generated heat above and beyond other thermal inputs. The heightened heat-inducing ability of such viscosity enhancement promotes entropy production in the system as a whole $\left( {N_{G} } \right)$. Figure 26 illustrated the elevated entropy layers, which improved the Brinkman number $\left( {B_{\varsigma } } \right)$ values. The primary feature of viscous dislocation heat produces a decrease in the escalating Brinkman numbers, which theoretically leads to a higher rate of entropy development.

## Final results and future guidance

The entropy production and heat transmission by a Prandtl–Eyring hybrid nanofluid (P-EHNF) over a stretched sheet is studied. By utilising a single model phase, a computational model may be developed. Several physical characteristics are used to derive the results. These include changes in velocity, energy, and entropy. Cattaneo–Christov heat flux $\varepsilon_{\varsigma }$ is also discussed in this study, as are the effects of Prandtl–Eyring parameters $A_{1}^{*}$ and $A_{2}^{*}$ as well as $K_{\varsigma }$ for porous medium, nanomolecular size $\phi$ and $\phi_{hnf}$, ${\Lambda }_{\varsigma }$ for velocity slip, thermal radiative variable $N_{\varsigma }$ and Biot number $H_{\varsigma }$ as well as various solid particle shapes $m$, $R_{e}$ and $B_{\varsigma }$. The following are the study's significant findings:

1. In comparison to traditional Prandtl–Eyring nanofluid (SWCNT/-EO), hybrid Prandtl–Eyring nanofluid (MWCNT-SWCNT/EO) is shown to be a superior thermal conductor.
2. Swelling the size of EO's nano solid particles can increase the rate of heat transmission.
3. Upsurges in the porous media variable $K$, the size parameters $\phi$ and $\phi_{hnf}$, thermal radiative flow ($N_{\varsigma }$), the Brinkman number ($B_{\varsigma }$) and the Reynolds number ($R_{e}$) growth the system's entropy, whereas the increase in the velocity slip parameter *(*$\Lambda_{\varsigma }$) reduces it.
4. Porous media variable increments enhance the velocity magnitude, whereas nano molecule swelling causes the speed to drop.

The current study's findings may help lead future heating system upgrades that evaluate the heating system's heat effect using a variety of non-Newtonian hybrid nanofluids (i.e., second-grade, Carreau, Casson, Maxwell, micropolar nanofluids, etc.). It's possible to depict the effects of temperature-dependent viscosity, temperature-dependent porosity, and magneto-slip flow by significantly expanding the scheme's capabilities.

## List of symbols

$A_{1}^{*}$: Prandtl–Eyring parameter-I
$A_{2}^{*}$: Prandtl–Eyring parameter-II
$$B_{\varsigma }$$: Brinkman number
$b$: Initial stretching rate
$$C_{f}$$: Drag force
$$C_{p}$$: Specific-heat $$({\text{J}}\,{\text{kg}}^{ - 1} \,{\text{K}}^{ - 1} )$$
$$E_{\varsigma }$$: Eckert number
*EO*: Engine Oil
$$E_{G}$$: Dimensional entropy $$({\text{J}}\,{\text{K}}^{ - 1} )$$
$$H_{\varsigma }$$: Biot number
$h_{\varsigma }$: Heat transfer coefficient
$k$: Porosity of fluid
$$\kappa$$: Thermal conductivity $$\left( {{\text{W}}\,{\text{m}}^{ - 1} \,K^{ - 1} } \right)$$
$k_{\varsigma }$: Thermal conductivity of the surface
$$k^{*}$$: Absorption coefficient
$K_{\varsigma }$: Porous media parameter
$$N_{\varsigma }$$: Radiation parameter
$N_{G}$: Dimensionless entropy generation
$Nu_{x}$: Local Nusselt number
$$P_{r}$$: Prandtl number $$(\nu /\alpha )$$
$p$: Column vectors of order $J \times 1$
$q_{r}$: Radiative heat flux
$q_{w}$: Wall heat flux
$$R_{e}$$: Reynolds number
$S$: Suction/injection parameter
$B_{1} ,B_{2}$: Velocity component in $x$, $y$ direction $({\text{m}}\,{\text{s}}^{ - 1} )$
$U_{w}$: The velocity of the stretching sheet
$$V_{\varsigma }$$: Vertical velocity
$x,y$: Dimensional space coordinates $({\text{m}})$

## Greek symbols

$$\yen$$: Fluid temperature
$\yen_{w}$: The fluid temperature of the surface
$$\yen_{\infty }$$: Ambient temperature
$\phi$: The volume fraction of the nanoparticles
$$\rho$$: Density ($${\text{kg}}\,{\text{m}}^{ - 3}$$)
$\sigma ^{*}$: Stefan Boltzmann constant
$$\psi $$: Stream function
$\Omega$: Independent similarity variable
$$\theta $$: Dimensionless temperature
$$\varepsilon_{\varsigma }$$: Relaxation time
${\Lambda }_{\varsigma }$: Velocity slip parameter
$\mu$: Dynamic viscosity of the fluid (${\text{kg}}\,{\text{m}}^{ - 1} \,{\text{s}}^{ - 1}$)
$\nu$: Kinematic viscosity of the fluid (${\text{m}}^{2} \,{\text{s}}^{ - 1}$)
$$\alpha$$: Thermal diffusivity $$({\text{m}}^{2} \,{\text{s}}^{ - 1} )$$
Π: Dimensionless temperature gradient

## Subscripts

$$f, gf$$: Base fluid
$$nf$$: Nanofluid
$$hnf$$: Hybrid nanofluid
$$p,p_{1} ,p_{2}$$: Nanoparticles
$$s$$: Particles
SWCNT: Single-walled carbon nanotubes
MWCNT: Multi-walled carbon nanotubes
